# Supplementary material for: DNA-PK and the TRF2 iDDR inhibit MRN-initiated resection at leading-end telomeres
Source: Nat Struct Mol Biol. 2023 Aug 31;30(9):1346–56. doi: 10.1038/s41594-023-01072-x (PMC10497418; doi:10.1038/s41594-023-01072-x)
Supplement: Supplementary file 1 — Reporting Summary [file 41594_2023_1072_MOESM1_ESM.pdf]

Corresponding author(s): DAPR NSMB-A47273

Last updated by author(s): Jul 8, 2023

Reporting Summary

Nature Portfolio wishes to improve the reproducibility of the work that we publish. This form provides structure for consistency and transparency in reporting. For further information on Nature Portfolio policies, see our Editorial Policies and the Editorial Policy Checklist.

Statistics

For all statistical analyses, confirm that the following items are present in the figure legend, table legend, main text, or Methods section.

n/a	Confirmed	
☐	☒	The exact sample size (*n*) for each experimental group/condition, given as a discrete number and unit of measurement
☐	☒	A statement on whether measurements were taken from distinct samples or whether the same sample was measured repeatedly
☐	☒	The statistical test(s) used AND whether they are one- or two-sided<br>*Only common tests should be described solely by name; describe more complex techniques in the Methods section.*
☒	☐	A description of all covariates tested
☐	☒	A description of any assumptions or corrections, such as tests of normality and adjustment for multiple comparisons
☐	☒	A full description of the statistical parameters including central tendency (e.g. means) or other basic estimates (e.g. regression coefficient) AND variation (e.g. standard deviation) or associated estimates of uncertainty (e.g. confidence intervals)
☐	☒	For null hypothesis testing, the test statistic (e.g. *F*, *t*, *r*) with confidence intervals, effect sizes, degrees of freedom and *P* value noted<br>*Give P values as exact values whenever suitable.*
☒	☐	For Bayesian analysis, information on the choice of priors and Markov chain Monte Carlo settings
☒	☐	For hierarchical and complex designs, identification of the appropriate level for tests and full reporting of outcomes
☒	☐	Estimates of effect sizes (e.g. Cohen's *d*, Pearson's *r*), indicating how they were calculated

Our web collection on statistics for biologists contains articles on many of the points above.

Software and code

Policy information about availability of computer code

Data collection	ChemiDoc (Bio-Rad), DeltaVision RT microscope system (GE Healthcare), Leica DMi8 microscope (Leica Microsystems) , Typhoon PhosphoImager (GE)
Data analysis	AlphaFold-Multimer (v2.1.0), Benchling [Biology Software] (2021), CellProfiler, Fiji (1.0), GraphPad Prism (v9 or 10.0.0), ImageQuant, Jalview (v2.11.2.7), Microsoft Excel (16.74), MUSCLE, NCBI MSA Viewer (v1.22.2), PyMol (v2.5.4), SnapGene (v4.3.11), SoftWoRX, UCSF ChimeraX (v1.2.5).

For manuscripts utilizing custom algorithms or software that are central to the research but not yet described in published literature, software must be made available to editors and reviewers. We strongly encourage code deposition in a community repository (e.g. GitHub). See the Nature Portfolio guidelines for submitting code & software for further information.

Data

Policy information about availability of data

All manuscripts must include a data availability statement. This statement should provide the following information, where applicable:

- Accession codes, unique identifiers, or web links for publicly available datasets
- A description of any restrictions on data availability
- For clinical datasets or third party data, please ensure that the statement adheres to our policy

All data generated or analyzed during this study are included in this article. Source data are provided with this paper. All other raw images are available from the corresponding authors upon reasonable request. Protein sequences were retreived from https://blast.ncbi.nlm.nih.gov/

Research involving human participants, their data, or biological material

Policy information about studies with human participants or human data. See also policy information about sex, gender (identity/presentation), and sexual orientation and race, ethnicity and racism.

Reporting on sex and gender	N/A
Reporting on race, ethnicity, or other socially relevant groupings	N/A
Population characteristics	N/A
Recruitment	N/A
Ethics oversight	N/A

Note that full information on the approval of the study protocol must also be provided in the manuscript.

Field-specific reporting

Please select the one below that is the best fit for your research. If you are not sure, read the appropriate sections before making your selection.

☒ Life sciences ☐ Behavioural & social sciences ☐ Ecological, evolutionary & environmental sciences

For a reference copy of the document with all sections, see nature.com/documents/nr-reporting-summary-flat.pdf

Life sciences study design

All studies must disclose on these points even when the disclosure is negative.

Sample size	No statistical method was used to predetermine sample size. Sample size was determined based on previous similar experiments: n=30-45 metaphases over 3-4 independent experiments for telomere fusions (Lottersberger, F., Karssemeijer, R. A., Dimitrova, N. & de Lange, T. 53BP1 and the LINC Complex Promote Microtubule-Dependent DSB Mobility and DNA Repair. Cell 163, 880–893 (2015)) and n=3-5 independent experiments for telomere overhang (Wu, P., van Overbeek, M., Rooney, S. & de Lange, T. Apollo contributes to G overhang maintenance and protects leading-end telomeres. Mol Cell 39, 606–617 (2010)).
Data exclusions	No data were excluded from the experiments in the study but metaphases were it was not possible to perform robust scoring (i.e. double CO-FISH staining, less than 10 chromosomes, all overlapping chromosomes) and nuclei with less than 10 telomere foci. In case of failed experiment (Cre or shRNA not working properly), all data associated with that specific experiment were not included.
Replication	The number of independent experiments performed is indicated in the figure legends. All sample images are representative of at least n=3 independent experiments with similar results, unless indicated.
Randomization	Randomization was done when possible. Culture dishes seeded at the same time with identical population were randomly chosen for the subsequent biological perturbation (Cre, shRNA, PARPi, TRF2 alleles). Pictures of metaphases and nuclei for all the cells/treatments were taken randomly by manual scanning of the slides/coverslips.
Blinding	Investigators were not blinded during the study. However, all samples were processed in parallel and treated identically for all the experiments. In most experiments blinded analysis is not applicable since the sample identity is readily apparent to the investigator.

Reporting for specific materials, systems and methods

We require information from authors about some types of materials, experimental systems and methods used in many studies. Here, indicate whether each material, system or method listed is relevant to your study. If you are not sure if a list item applies to your research, read the appropriate section before selecting a response.

Materials & experimental systems

n/a	Involved in the study
☐	☒ Antibodies
☐	☒ Eukaryotic cell lines
☒	☐ Palaeontology and archaeology
☐	☒ Animals and other organisms
☒	☐ Clinical data
☒	☐ Dual use research of concern
☒	☐ Plants

Methods

n/a	Involved in the study
☒	☐ ChIP-seq
☒	☐ Flow cytometry
☒	☐ MRI-based neuroimaging

Antibodies

Antibodies used	Immuniblot: beta-Actin (#3700; Cell Signal, 1:1000); Chk2 (BD 611570; BD Biosciences, 1:800); DNA-PKcs (SC-1552; Santa Cruz Biotechnology, 1:200); Ku70 (sc-17789 or sc-1487; Santa Cruz Biotechnology, 1:200); Lig3 (SC-135883; Santa Cruz Biotechnology, 1:1000); Nbs1 (ab175800; Abcam, 1:1000); TRF2 (#13136; Cell Signal, 1:500); gamma-Tubulin (GTU-88; GeneTex, 1:1000); and secondary anti-Mouse/anti-Rabbit IgG HRP (Cytiva).<br>Immunofluorescence: gamma-H2AX (JBW301, Millipore; 1:1000) primary antibodies, and secondary anti-mouse AlexaFluor 647 antibody (A32728, Invitrogen),
Validation	Antibodies against beta-Actin, Chk2, Lig3, Nbs1, TRF2 and gamma-Tubulin were validated by the suppliers companies for reactivity against the mouse proteins by Immunoblot. Antibodies against gamma-H2AX were validated by the suppliers companies for reactivity against the mouse proteins by Immunofluorescence.<br>Antibodies against DNA-PKcs and Ku70 were validated by the suppliers companies for reactivity against the human proteins. We validated them for reactivity against the mouse proteins by the Immunoblot in Extended Data Fig1 on genotyped MEFs.

Eukaryotic cell lines

Policy information about cell lines and Sex and Gender in Research

Cell line source(s)	293T/17 [HEK 293T/17] (CRL-11268) and Phoenix ECO cells (CRL-3214) were obtained by ATCC, Rockville, MD).<br>MEFs used in this study were generated previously (Dimitrova, N. & de Lange, T. Cell cycle-dependent role of MRN at dysfunctional telomeres: ATM signaling-dependent induction of nonhomologous end joining (NHEJ) in G1 and resection-mediated inhibition of NHEJ in G2. Mol Cell Biol 29, 5552–5563 (2009); Lottersberger, F., Karssemeijer, R. A., Dimitrova, N. & de Lange, T. 53BP1 and the LINC Complex Promote Microtubule-Dependent DSB Mobility and DNA Repair. Cell 163, 880–893 (2015); Wu, P., van Overbeek, M., Rooney, S. & de Lange, T. Apollo contributes to G overhang maintenance and protects leading-end telomeres. Mol Cell 39, 606–617 (2010)) or for this study by F.L, K.T. or P.W. in T.d.L. laboratory.
Authentication	No authentication was performed for 293T and Phoenix ECO cells.<br>MEFs were genotyped by Transnetyx Inc. using real-time PCR and authenticated when possible by Immunoblots (for DNA-PKcs, Ku70 and TRF2 deletion).
Mycoplasma contamination	All cells tested negative for Mycoplasma contamination
Commonly misidentified lines<br>(See ICLAC register)	No commonly misidentified cell lines were used in this study.

Animals and other research organisms

Policy information about studies involving animals; ARRIVE guidelines recommended for reporting animal research, and Sex and Gender in Research

Laboratory animals	Pregnant female mice were used to isolate MEFs from E12.5 embryos.<br>Species Mus musculus musculus;; Strain mixed C57BL/6 and 129 ; sex female and male; age range 2-10 months.
Wild animals	N/A
Reporting on sex	N/A
Field-collected samples	N/A
Ethics oversight	Mice were housed and cared for under the Rockefeller University AIACUC protocol 22030-H at the Rockefeller University's Comparative Bioscience Center, which provides animal care according to NIH guidelines.

Note that full information on the approval of the study protocol must also be provided in the manuscript.
