# Supplementary material for: DNA-PK and the TRF2 iDDR inhibit MRN-initiated resection at leading-end telomeres
Source: Nat Struct Mol Biol. 2023 Aug 31;30(9):1346–56. doi: 10.1038/s41594-023-01072-x (PMC10497418; doi:10.1038/s41594-023-01072-x)

Source Data Fig.1
Fig.1a, b
Blue: DAPI (DNA)
Red: Cy3-OO-(CCCTAA)$_3$ (Lagging-end telomeres)
Green: Alexa-488 -(TTAGGG)$_3$ (Leading-end telomeres)
100 pixel = 10.8 μm

Apollo$^{F/F}$ DNA-PKcs$^{+/+}$ Ku70$^{+/+}$ no Cre:

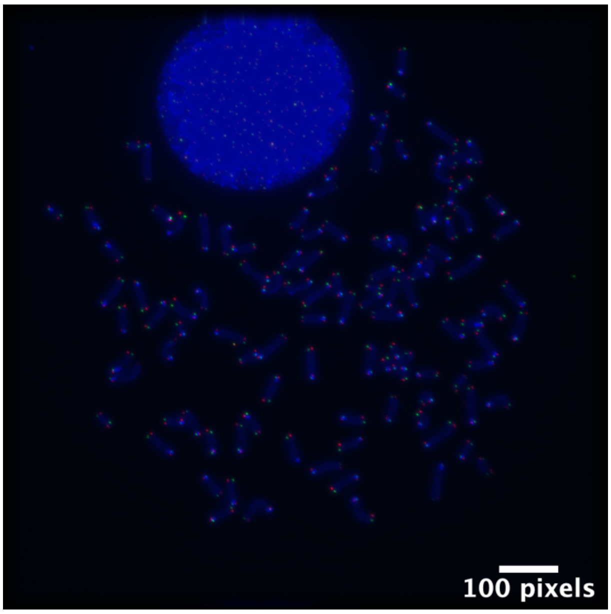

Apollo$^{F/F}$ DNA-PKcs$^{+/+}$ Ku70$^{+/+}$ + Cre:

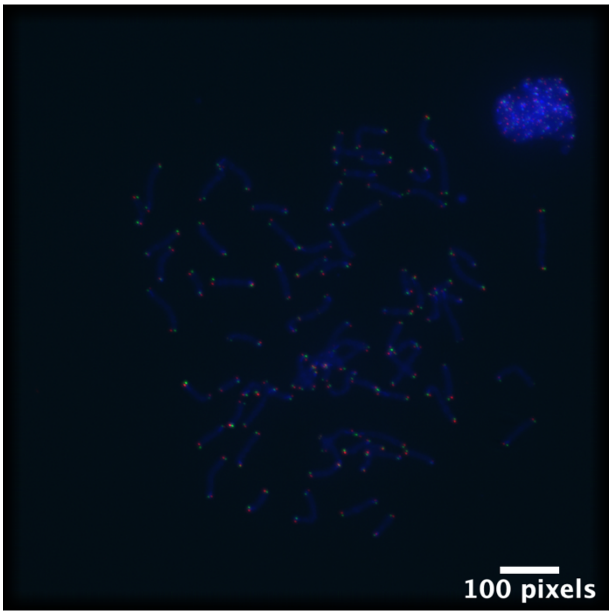

Apollo$^{F/F}$ DNA-PKcs$^{+/+}$ Ku70$^{-/-}$ no Cre:

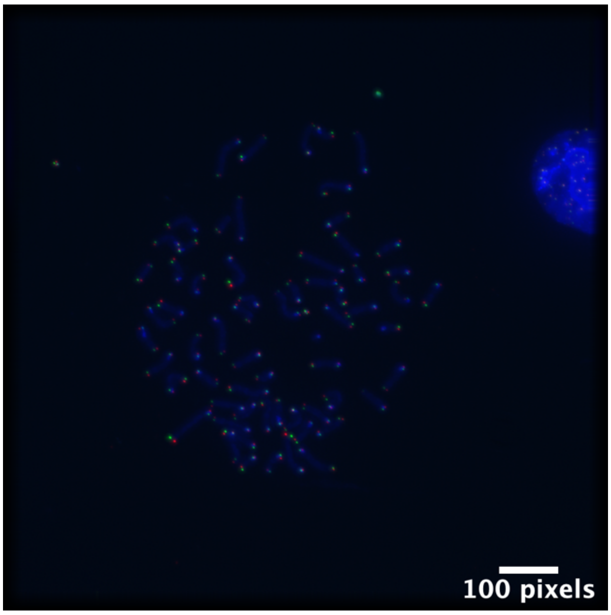

Apollo$^{F/F}$ DNA-PKcs$^{+/+}$ Ku70$^{-/-}$ + Cre:

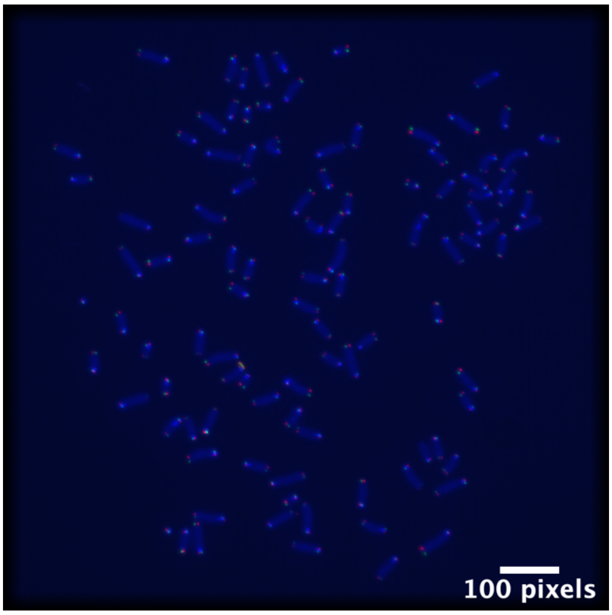

Apollo$^{F/F}$ DNA-PKcs$^{-/-}$ Ku70$^{+/+}$ no Cre:

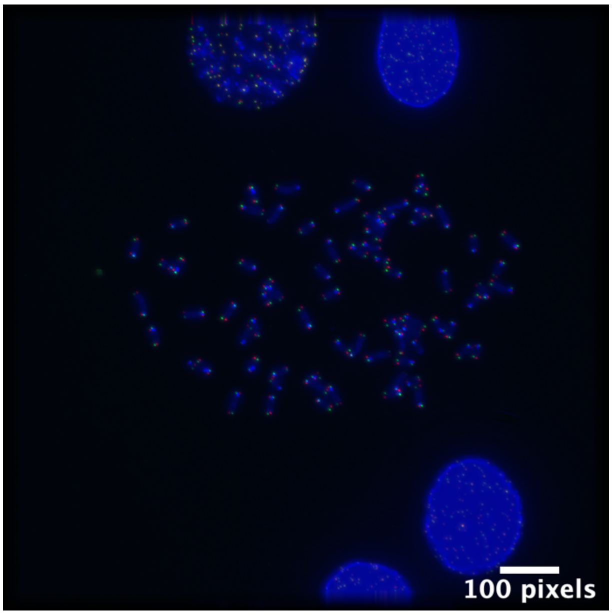

Apollo$^{F/F}$ DNA-PKcs$^{-/-}$ Ku70$^{+/+}$ + Cre:

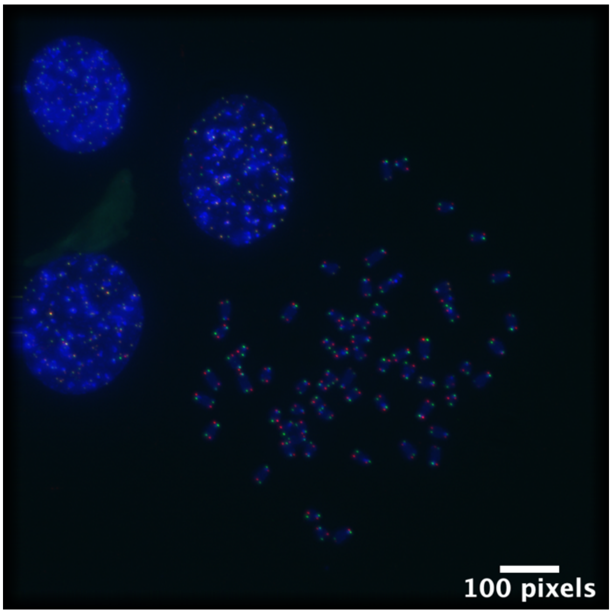

Apollo$^{F/F}$ DNA-PKcs$^{-/-}$ Ku70$^{-/-}$ no Cre:

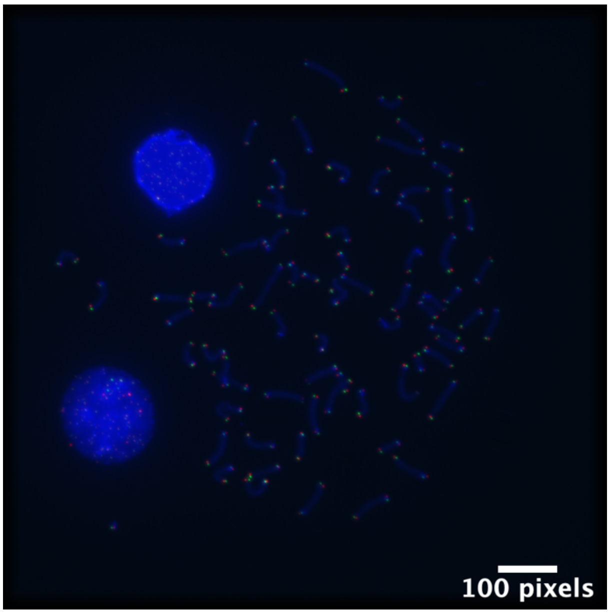

Apollo$^{F/F}$ DNA-PKcs$^{-/-}$ Ku70$^{-/-}$ + Cre:

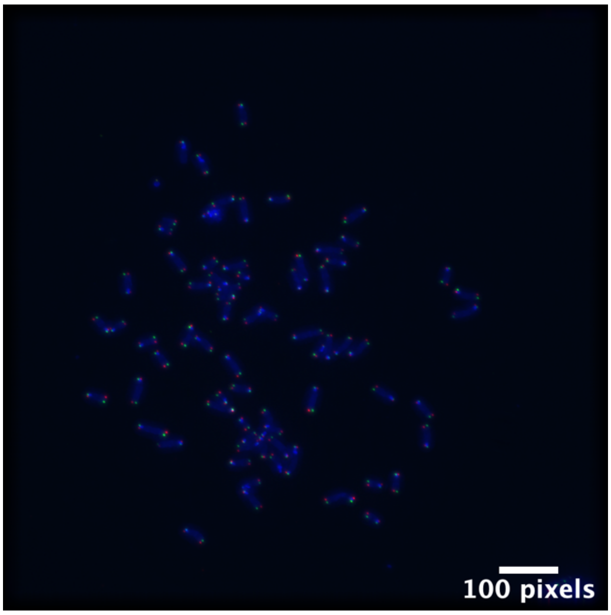

Fig.1c, d:
Blue: DAPI (DNA)
Red: Cy3-OO-$(CCCTAA)_3$ (Lagging-end telomeres)
Green: Alexa-488 -$(TTAGGG)_3$ (Leading-end telomeres)
100 pixel = 10.8 μm

Apollo$^{F/F}$ Lig4$^{-/-}$ no Cre:

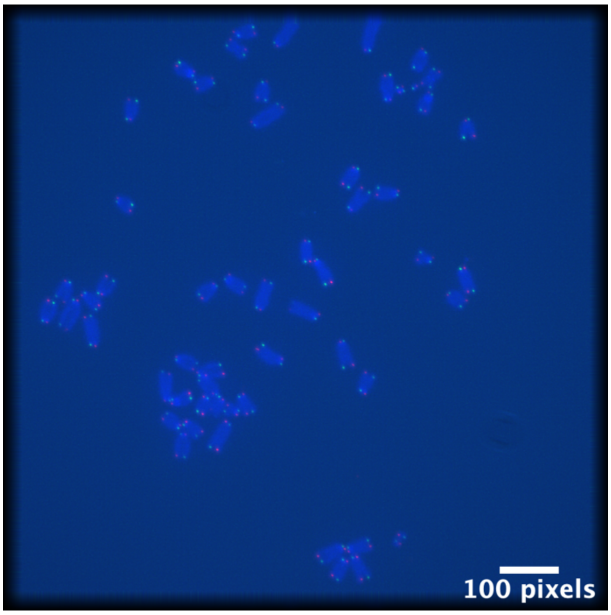

Apollo$^{F/F}$ Lig4$^{-/-}$ + Cre:

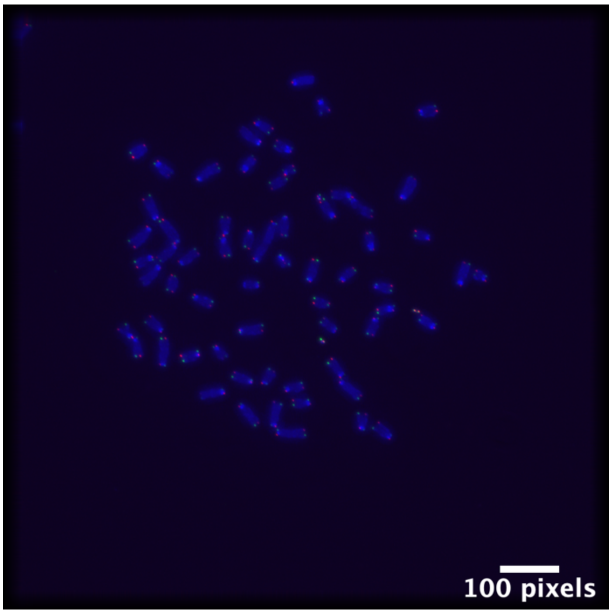

Fig.1e,f:
Blue: DAPI (DNA)
Red: Cy3-OO-$(CCCTAA)_3$ (Lagging-end telomeres)
Green: Alexa Fluor 488-OO-$(TTAGGG)_3$ (Leading-end telomeres)
100 pixel = 10.8 μm

ApolloF/F + Cre:

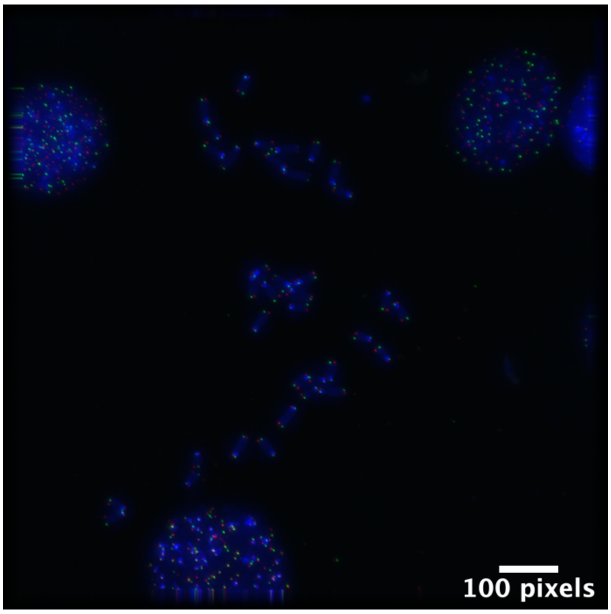

ApolloF/F + Cre + PARPi:

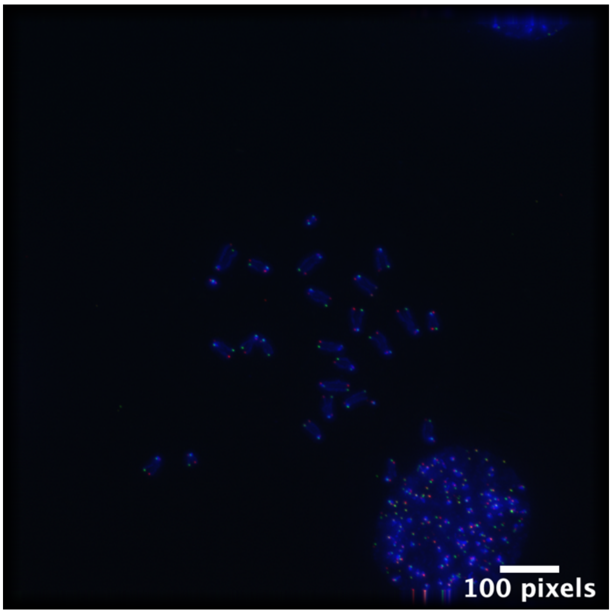

Fig.1g, h:
Blue: DAPI (DNA)
Red: Cy3-OO-(CCCTAA)$_3$ (Lagging-end telomeres)
Green: Alexa Fluor 488-OO-(TTAGGG)$_3$ (Leading-end telomeres)

ApolloF/F no shRNA + Cre:

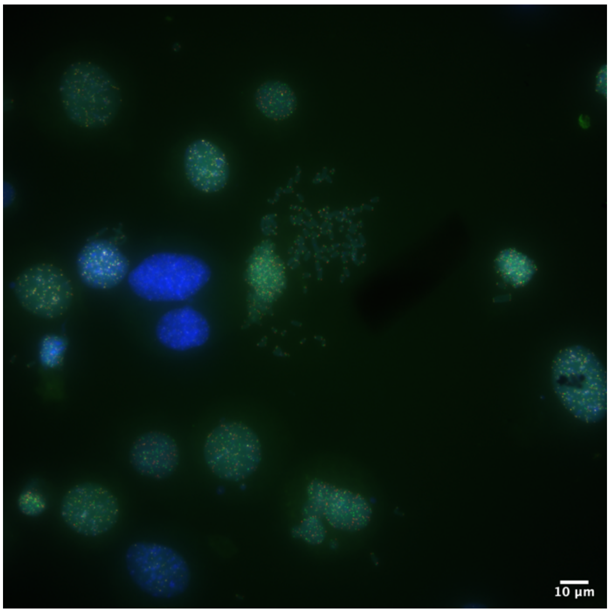

ApolloF/F shLig3 + Cre:

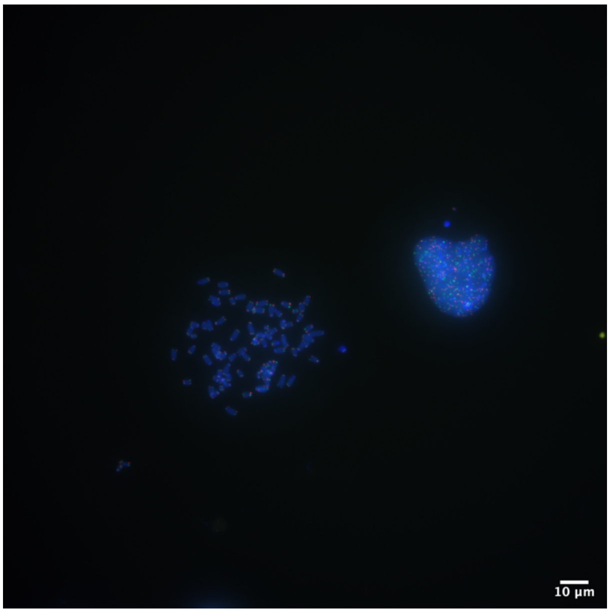

ApolloF/F shPolQ + Cre:

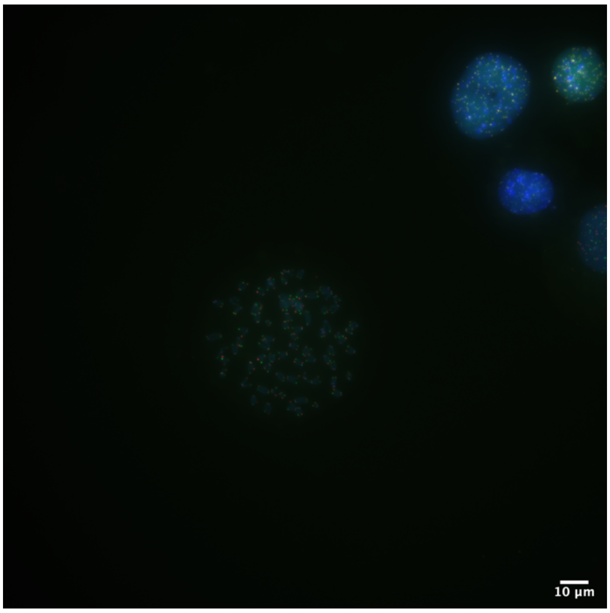

Supplement: Source Data Fig. 1 — Uncropped and unprocessed metaphases. [file 41594_2023_1072_MOESM3_ESM.pdf]
