# Supplementary material for: DNA-PK and the TRF2 iDDR inhibit MRN-initiated resection at leading-end telomeres
Source: Nat Struct Mol Biol. 2023 Aug 31;30(9):1346–56. doi: 10.1038/s41594-023-01072-x (PMC10497418; doi:10.1038/s41594-023-01072-x)
Supplement: Source Data Fig. 2 — Uncropped scans of telomere overhang gels. [file 41594_2023_1072_MOESM5_ESM.pdf]

Figure 2

Figure 2a-b: IV A

Native: 1 2 3 4 5 6 7 8 9 10 11 12

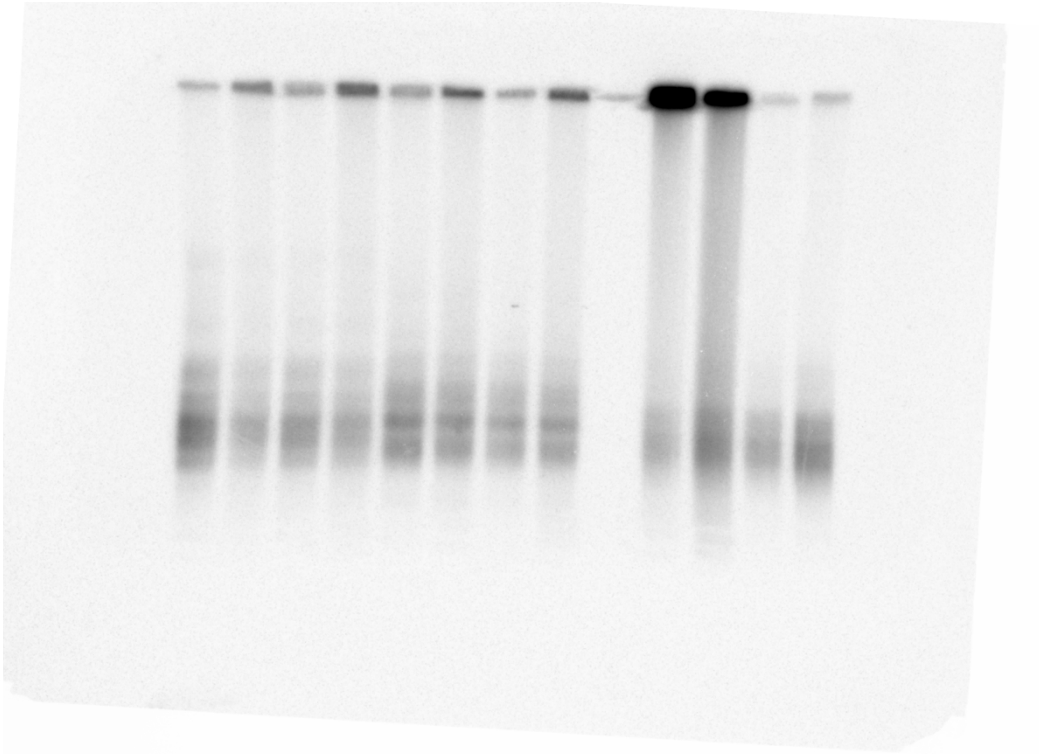

Denatured: 1 2 3 4 5 6 7 8 9 10 11 12

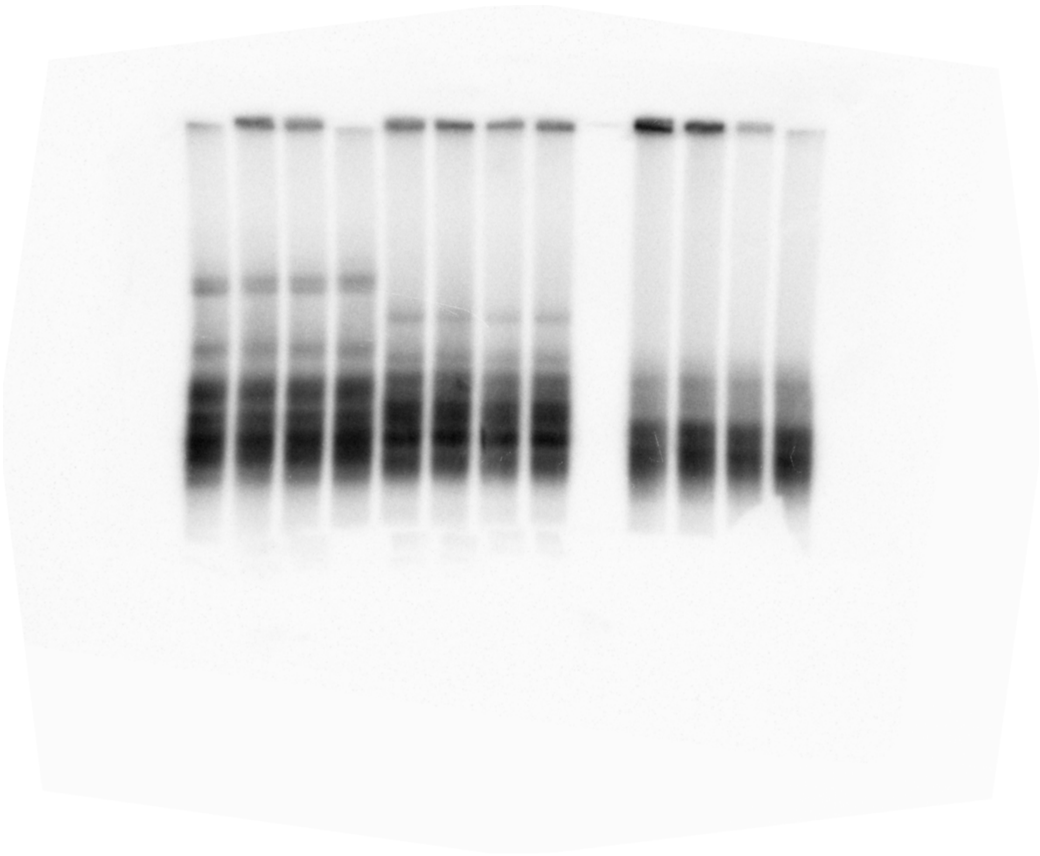

1. Apollo$^{F/F}$ DNAPKcs$^{+/+}$ Ku70$^{+/+}$ no Cre
2. Apollo$^{F/F}$ DNAPKcs$^{+/+}$ Ku70$^{+/+}$ + Cre
3. /
4. /
5. Apollo$^{F/F}$ DNAPKcs$^{-/-}$ Ku70$^{+/+}$ no Cre
6. Apollo$^{F/F}$ DNAPKcs$^{-/-}$ Ku70$^{+/+}$ + Cre
7. /
8. /
9. /
10. /
11. /
12. /

Figure 2a-b: IV B

Native: 2 3 4 5 6 7 8 9 10 11 12 13 14

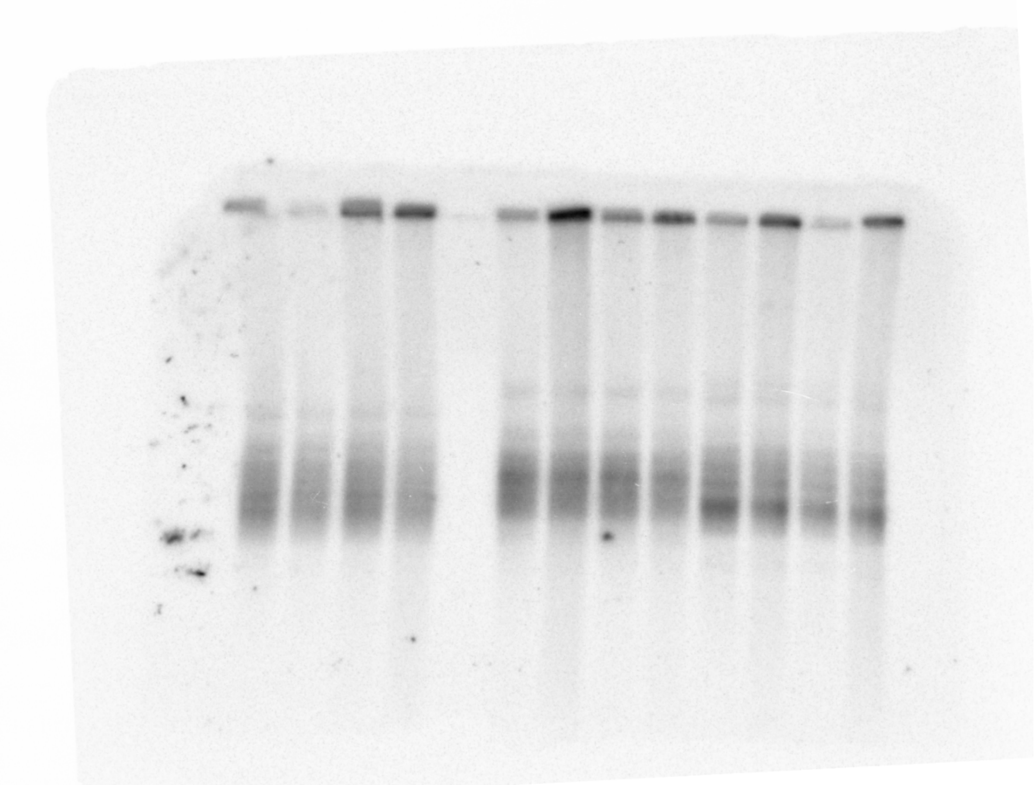

Denatured: 2 3 4 5 6 7 8 9 10 11 12 13 14

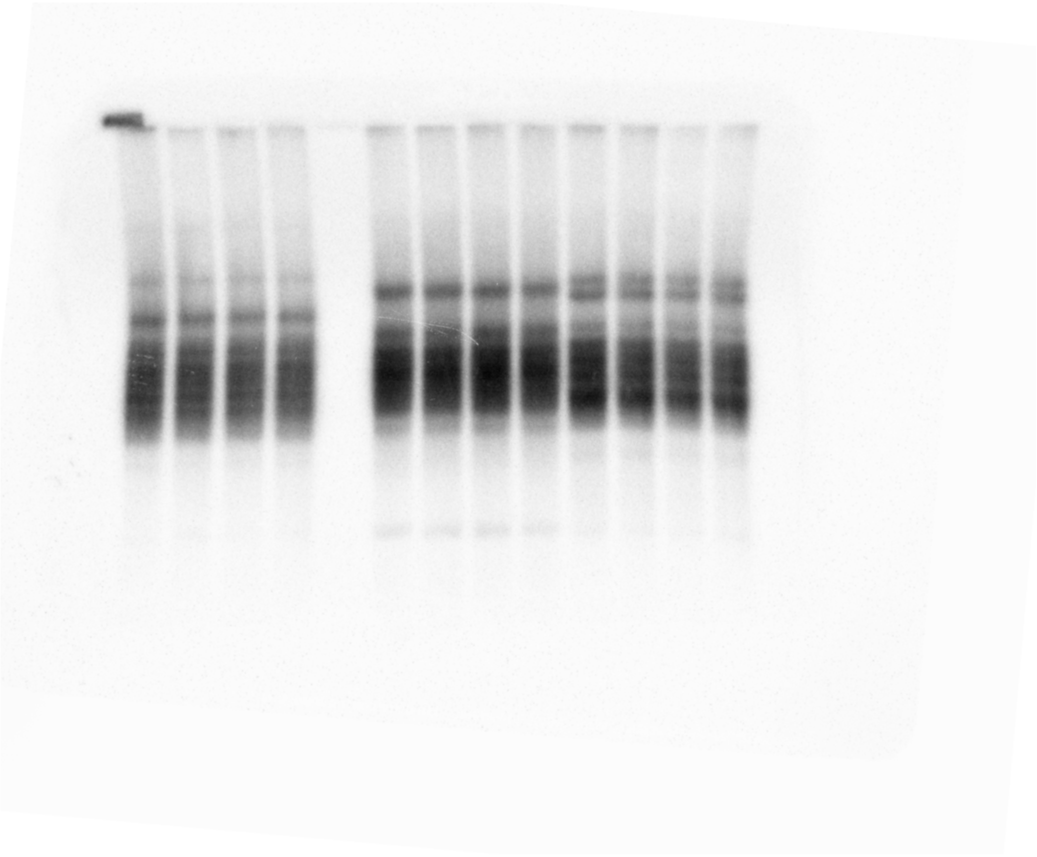

2. /
3. /
4. /
5. /
6. /
7. Apollo$^{F/F}$ DNAPKcs$^{+/+}$ Ku70$^{-/-}$ no Cre
8.Apollo$^{F/F}$ DNAPKcs$^{+/+}$ Ku70$^{-/-}$ + Cre
9. /
10. /
11. Apollo$^{F/F}$ DNAPKcs$^{-/-}$ Ku70$^{-/-}$ no Cre
12. Apollo$^{F/F}$ DNAPKcs$^{-/-}$ Ku70$^{-/-}$ + Cre
13. /
14. /

Figure 2c-d: experiment I

Native: 1 2 3 4 5 6

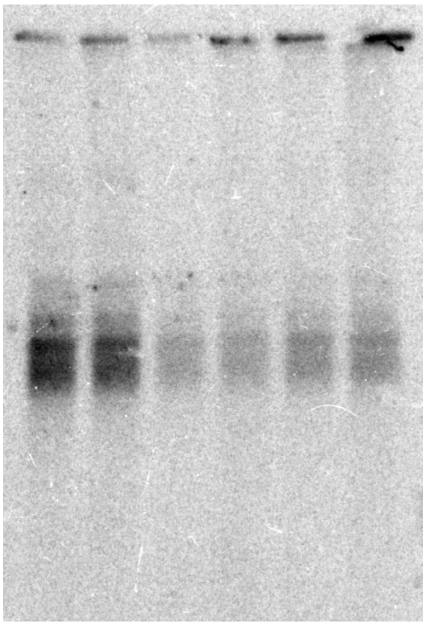

Denatured: 1 2 3 4 5 6

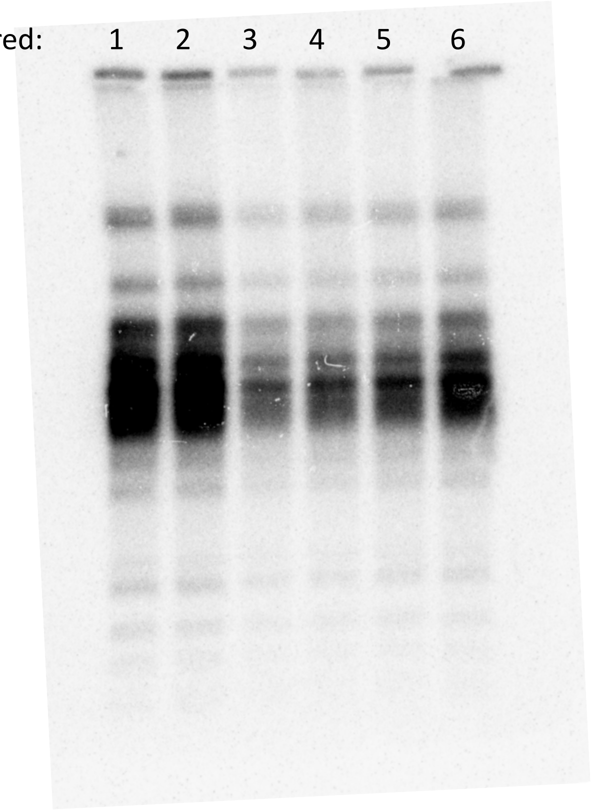

1. Apollo$^{F/F}$ vec no Cre
2. Apollo$^{F/F}$ vec plus Cre
3. Apollo$^{F/F}$ shLig3 no Cre
4. Apollo$^{F/F}$ shLig3 plus Cre
5. Apollo$^{F/F}$ shPolQ no Cre
6. Apollo$^{F/F}$ shPolQ plus Cre

Figure 2e-f: experiment I

Native: 1 2 3 4 5 6

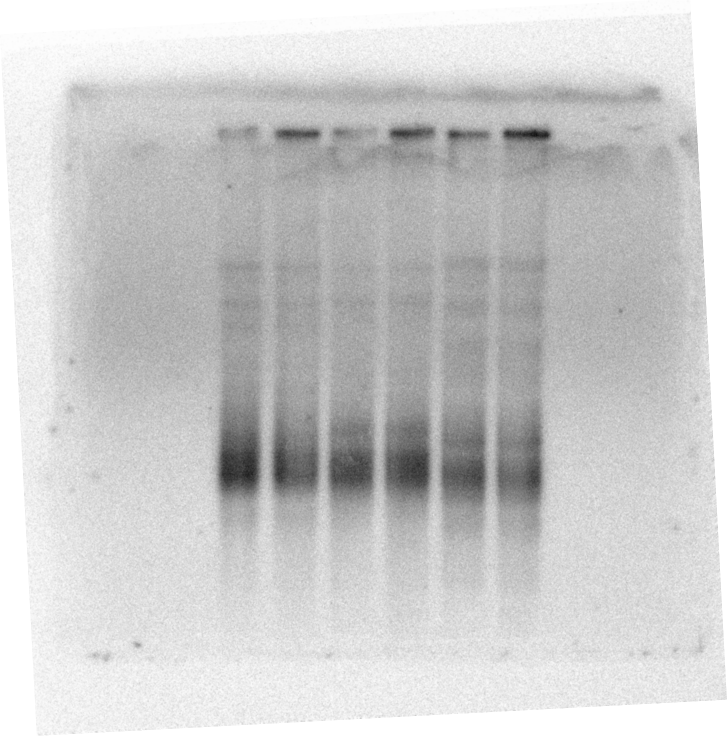

Denatured: 1 2 3 4 5 6

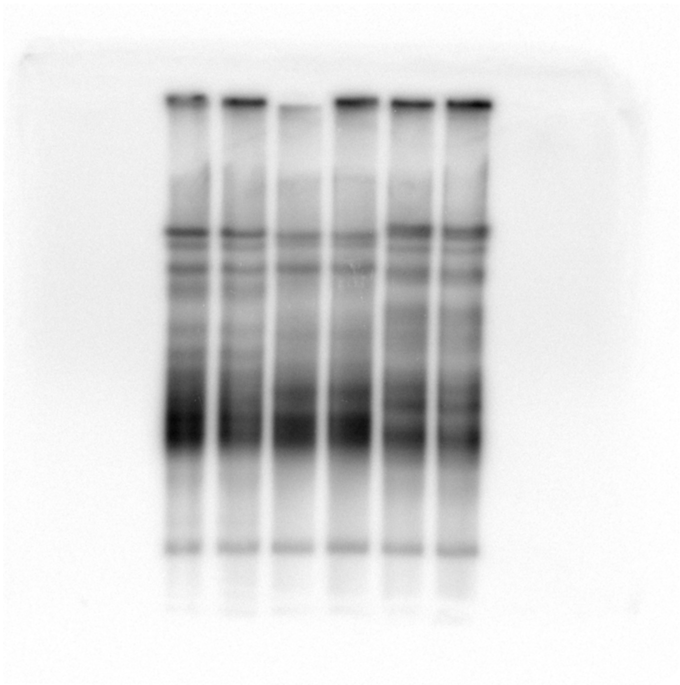

7. Ku70$^{F/+}$ no Cre
8. Ku70$^{F/+}$ + Cre
9. Ku70$^{F/F}$ no Cre
10. Ku70$^{F/F}$ + Cre
11. /
12. /
