# Supplementary material for: DNA-PK and the TRF2 iDDR inhibit MRN-initiated resection at leading-end telomeres
Source: Nat Struct Mol Biol. 2023 Aug 31;30(9):1346–56. doi: 10.1038/s41594-023-01072-x (PMC10497418; doi:10.1038/s41594-023-01072-x)

Source Data Fig.3
Fig.3a

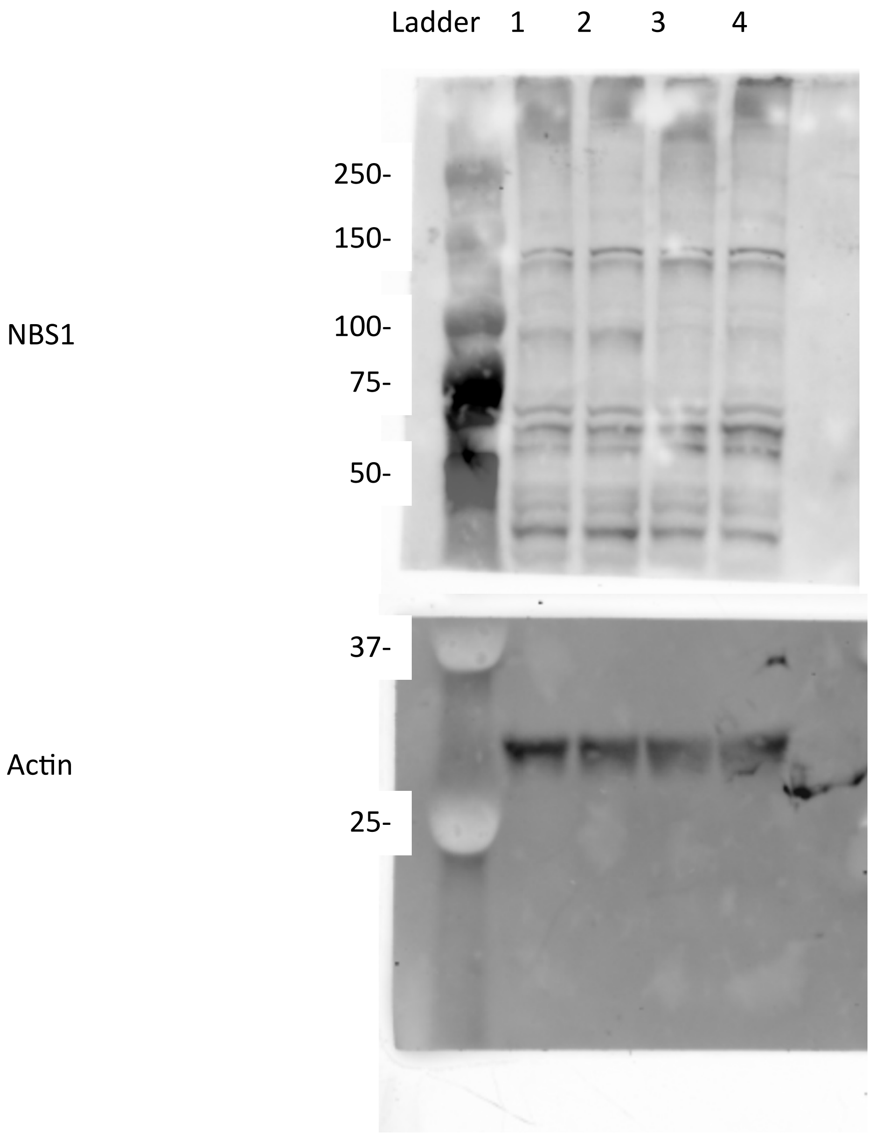

Apollo$^{F/F}$ DNA-PKcs$^{-/-}$:

1. EV no Cre
2. EV + Cre
3. sgNBS1 no Cre
4. sgNBS1 + Cre

Fig.3b
Blue: DAPI (DNA)
Red: Cy3-OO-$(CCCTAA)_3$ (Lagging-end telomeres)
Green: Alexa Fluor 488-OO-$(TTAGGG)_3$ (Leading-end telomeres)

Apollo$^{F/F}$ DNA-PKcs$^{-/-}$ no sgRNA no Cre:

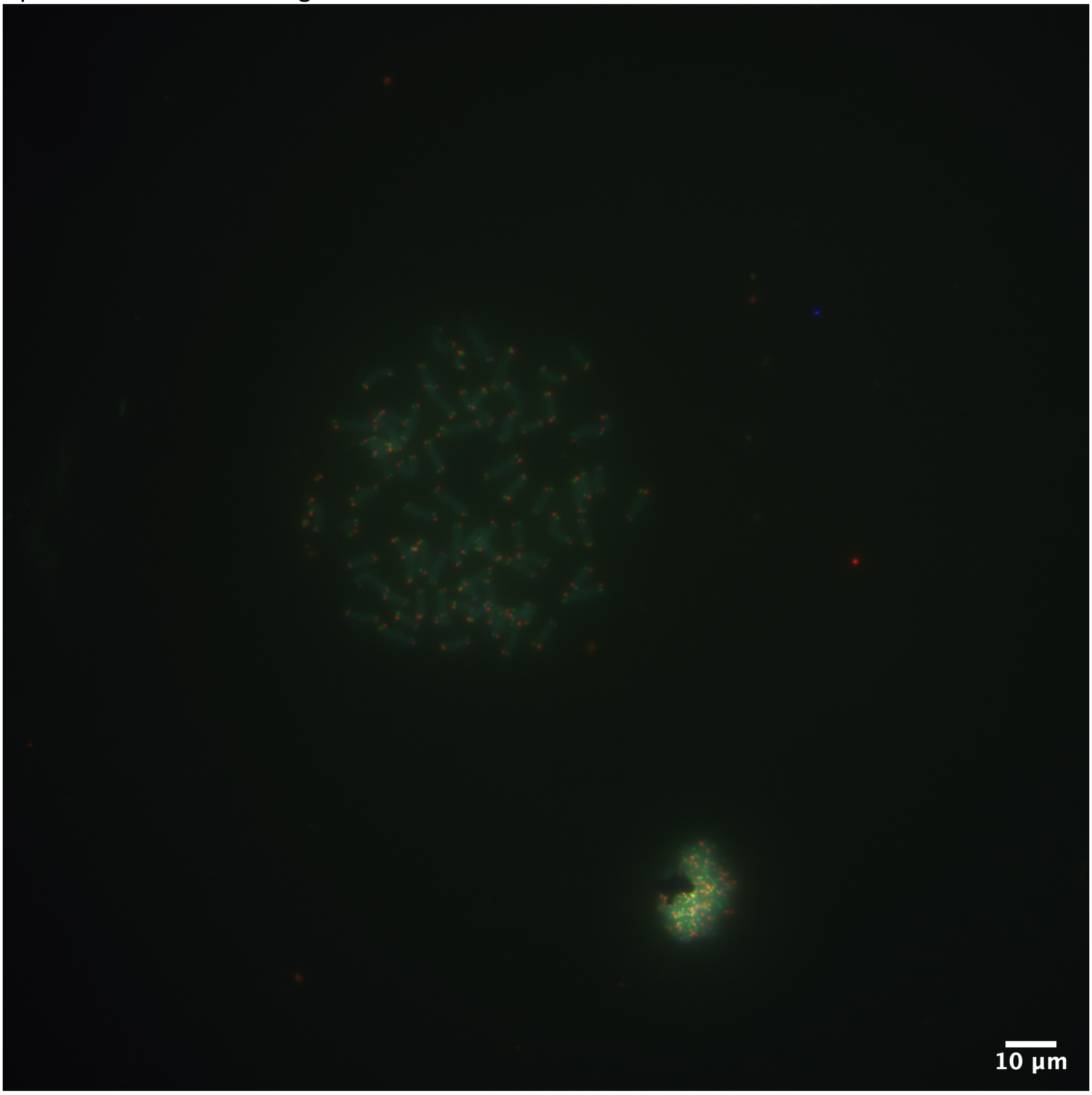

Apollo$^{F/F}$ DNA-PKcs$^{-/-}$ no sgRNA + Cre:

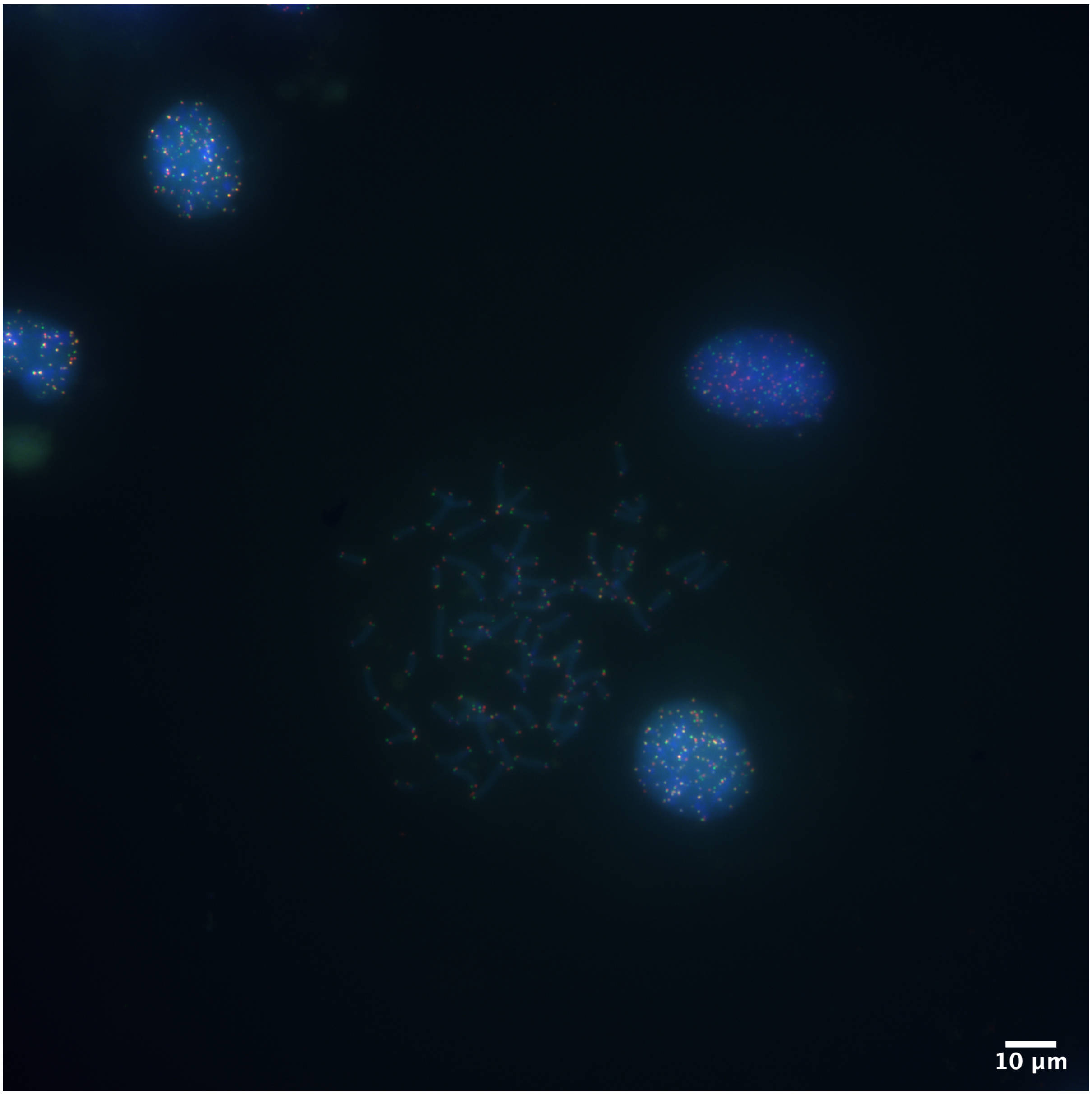

Apollo$^{F/F}$ DNA-PKcs$^{-/-}$ + sgNBS1 no Cre:

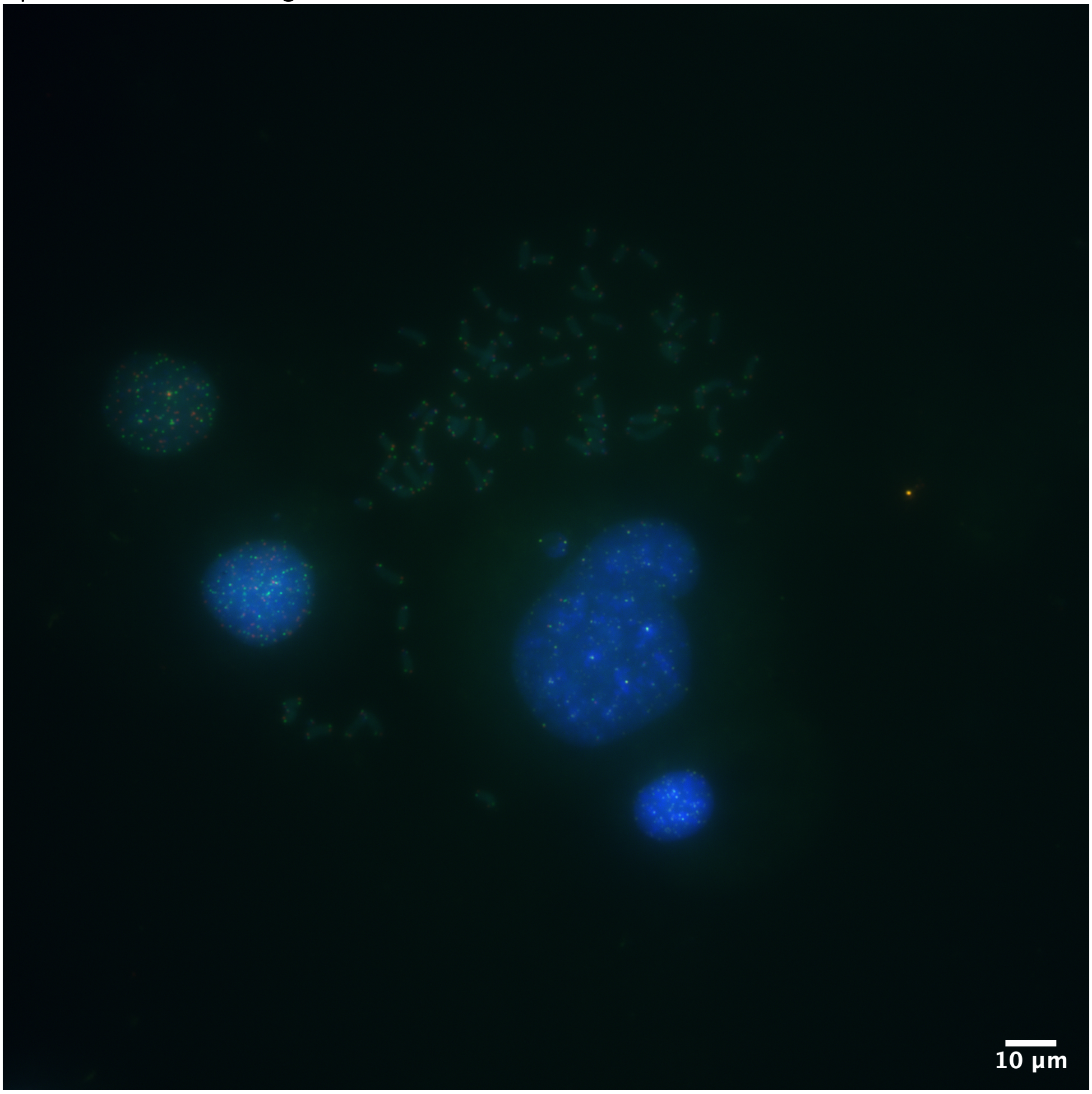

Apollo$^{F/F}$ DNA-PKcs$^{-/-}$ + sgNBS1 + Cre:

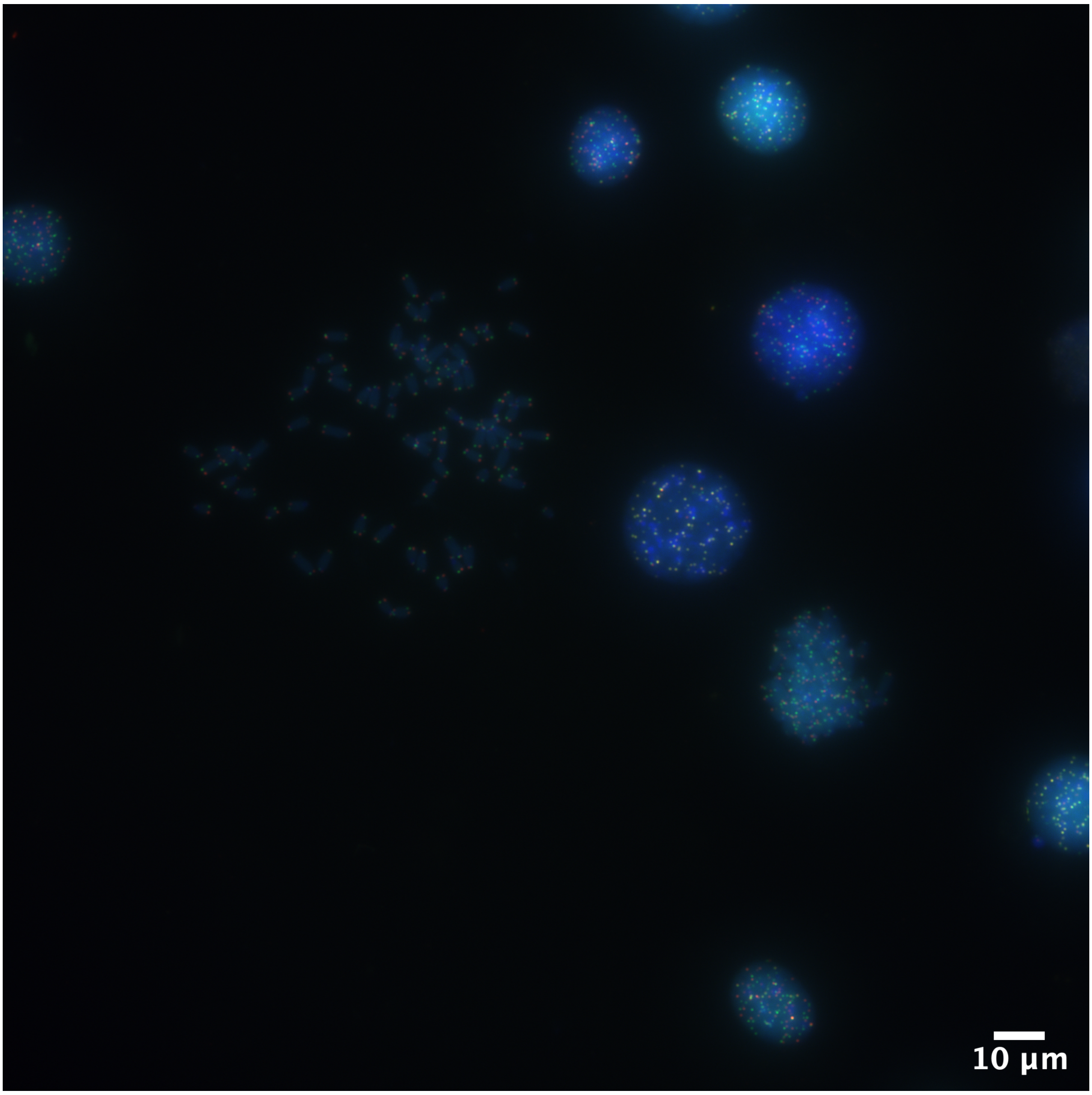

Supplement: Source Data Fig. 3 — Uncropped western blots. Uncropped and unprocessed metaphases. [file 41594_2023_1072_MOESM7_ESM.pdf]
