# Supplementary material for: DNA-PK and the TRF2 iDDR inhibit MRN-initiated resection at leading-end telomeres
Source: Nat Struct Mol Biol. 2023 Aug 31;30(9):1346–56. doi: 10.1038/s41594-023-01072-x (PMC10497418; doi:10.1038/s41594-023-01072-x)

Fig. 4

Fig. 4a

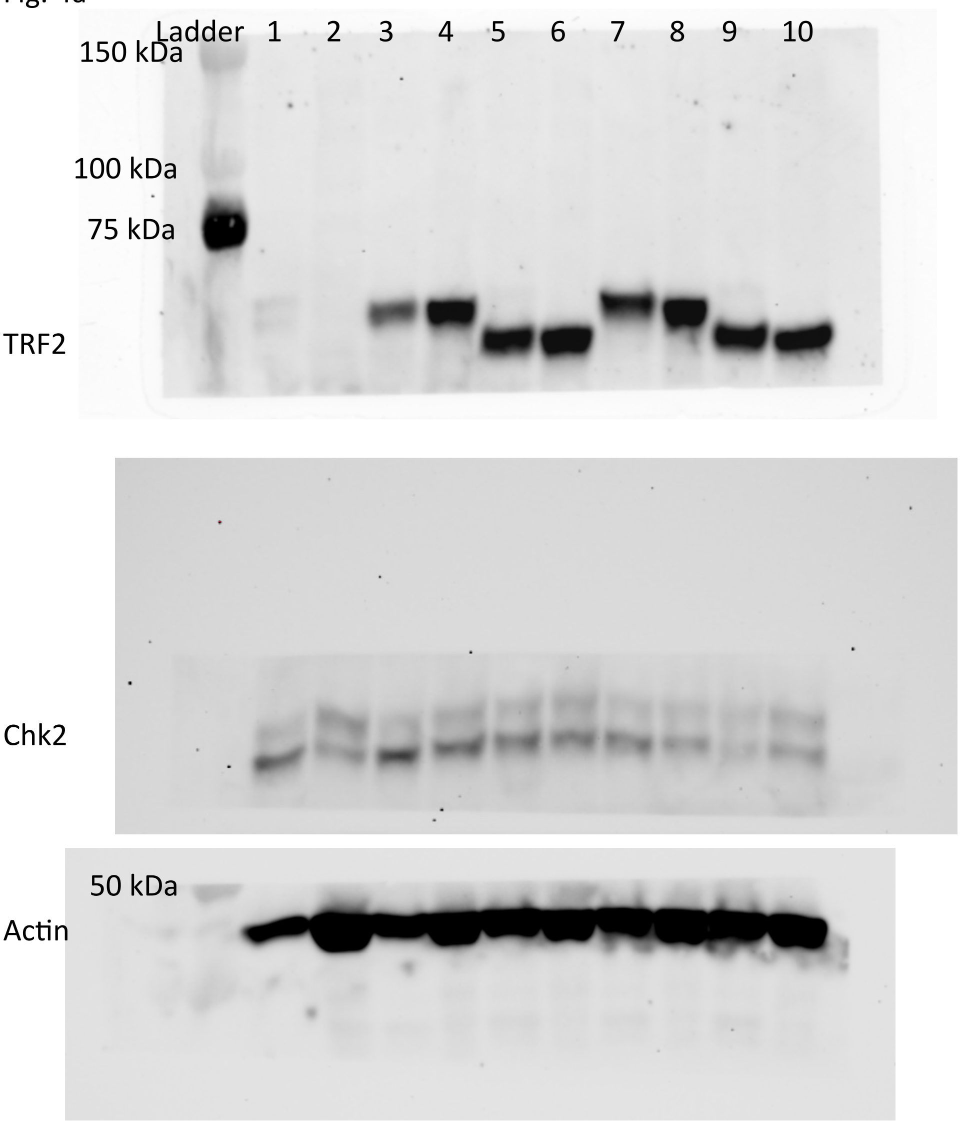

TRF2F/F Rosa-CreER^{T1} +

1. vec no Cre
2. vec + Cre
3. TRF2 no Cre
4. TRF2 + Cre
5. TRF2-ΔiDDR no Cre
6. TRF2-ΔiDDR + Cre
7. TRF2-F120A no Cre
8. TRF2-F120A + Cre
9. TRF2-F120AΔiDDR no Cre
10. TRF2-F120AΔiDDR + Cre

Fig. 4b,c: experiment 2

Native: 1 2 3 4 5 6 7 8 9 10

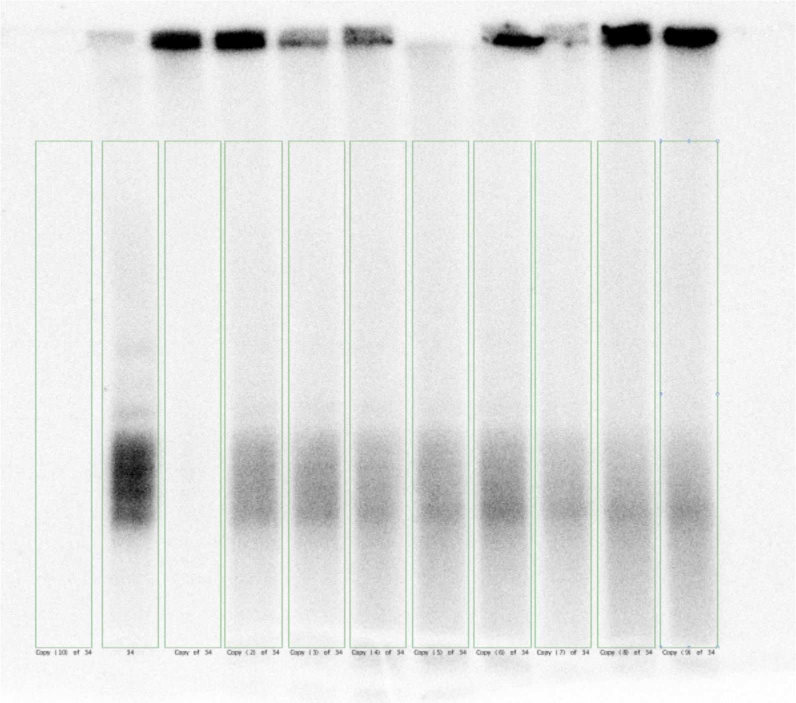

TRF2F/F Rosa-CreER^{T1} +

1. vec no 4-OHT
2. vec + 4-OHT
3. TRF2 no 4-OHT
4. TRF2 + 4-OHT
5. TRF2-ΔiDDR no 4-OHT
6. TRF2-ΔiDDR + 4-OHT
7. TRF2-F120A no 4-OHT
8. TRF2-F120A + 4-OHT
9. TRF2-F120AΔiDDR no - 4-OHT
10. TRF2-F120AΔiDDR + 4-OHT

Denatured: 1 2 3 4 5 6 7 8 9 10

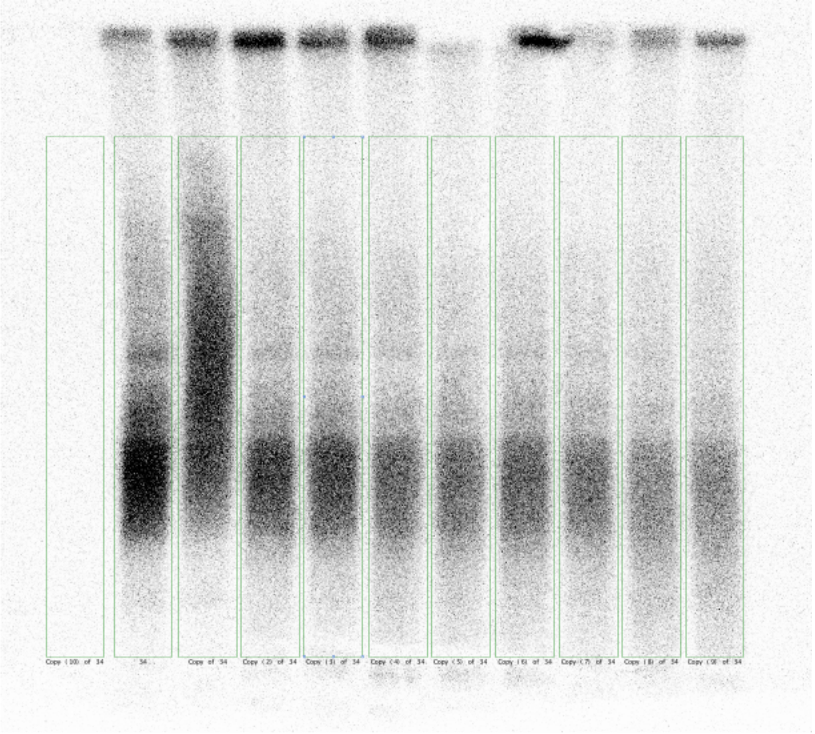

Figure 4d,e:
Blue: DAPI (DNA)
Red: Cy3-OO-$(TTAGGG)_3$ (Leading-end telomeres)
Far-red/changed to green: Alexa Fluor 647-OO-$(CCCTAA)_3$ (Lagging-end telomeres)

TRF2F/F Rosa-CreER^{T1} + vec + 4-OHT

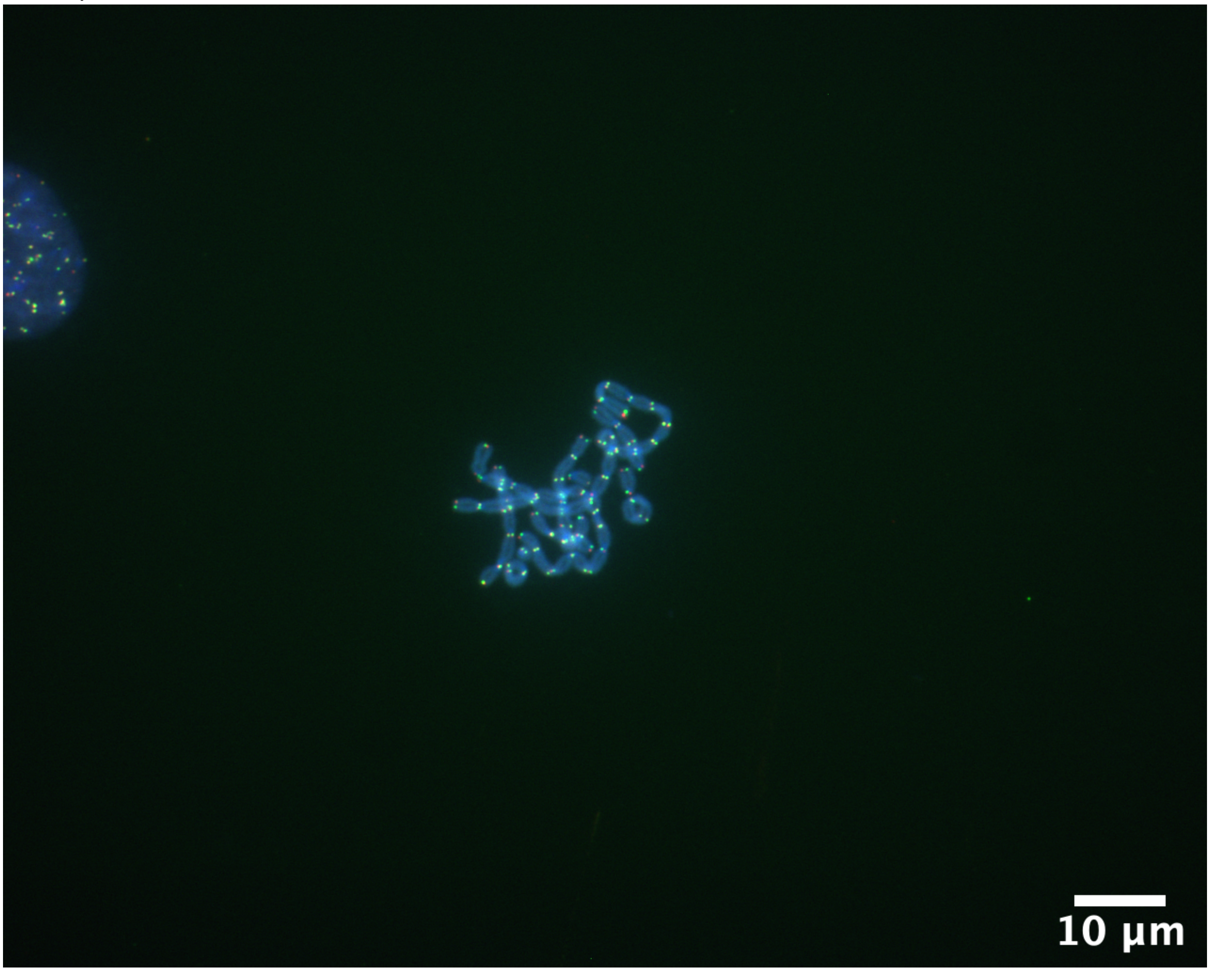

TRF2F/F Rosa-CreER^{T1} + TRF2-WT + 4-OHT

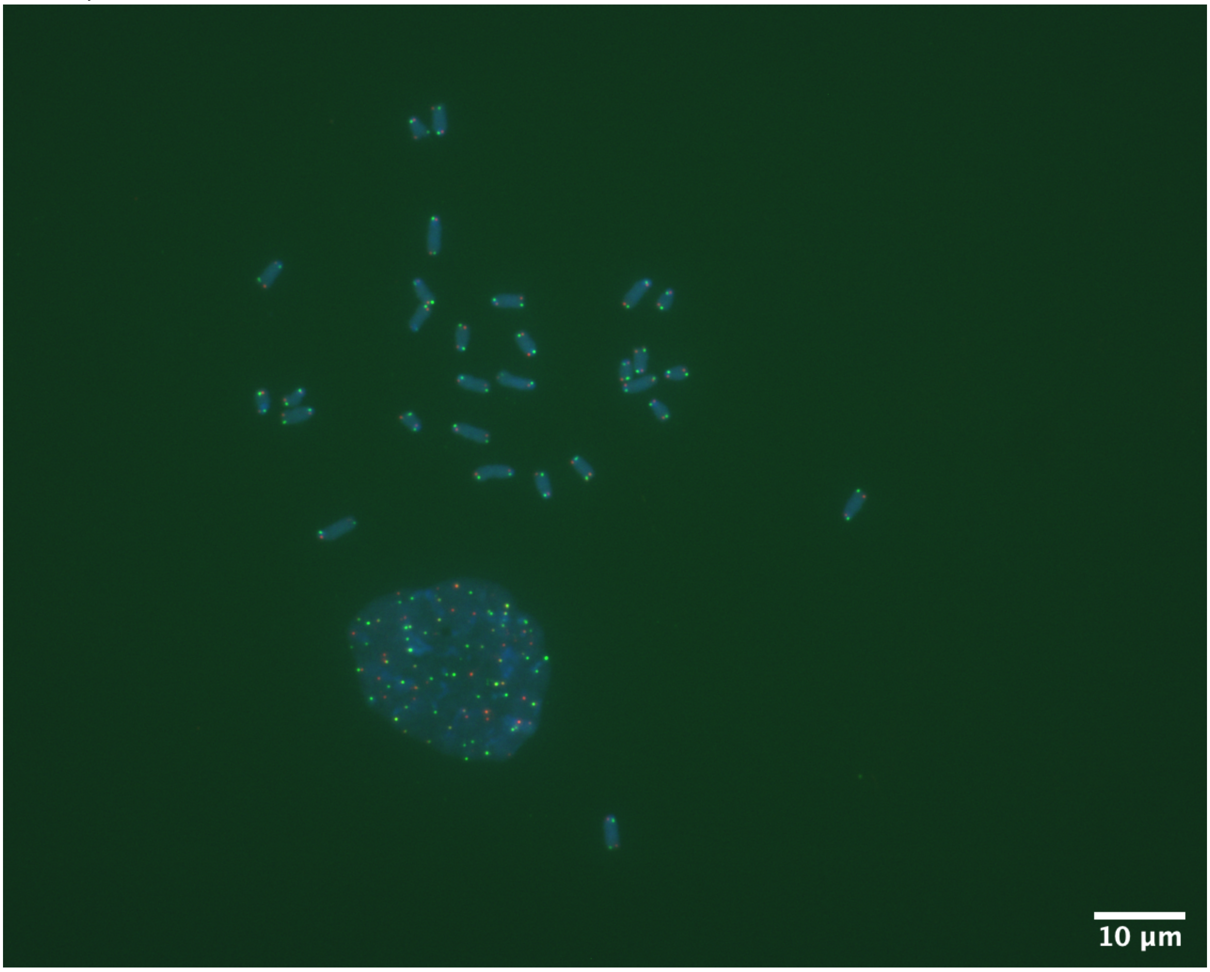

TRF2F/F Rosa-CreER^{T1} + TRF2-∆iDDR + 4-OHT

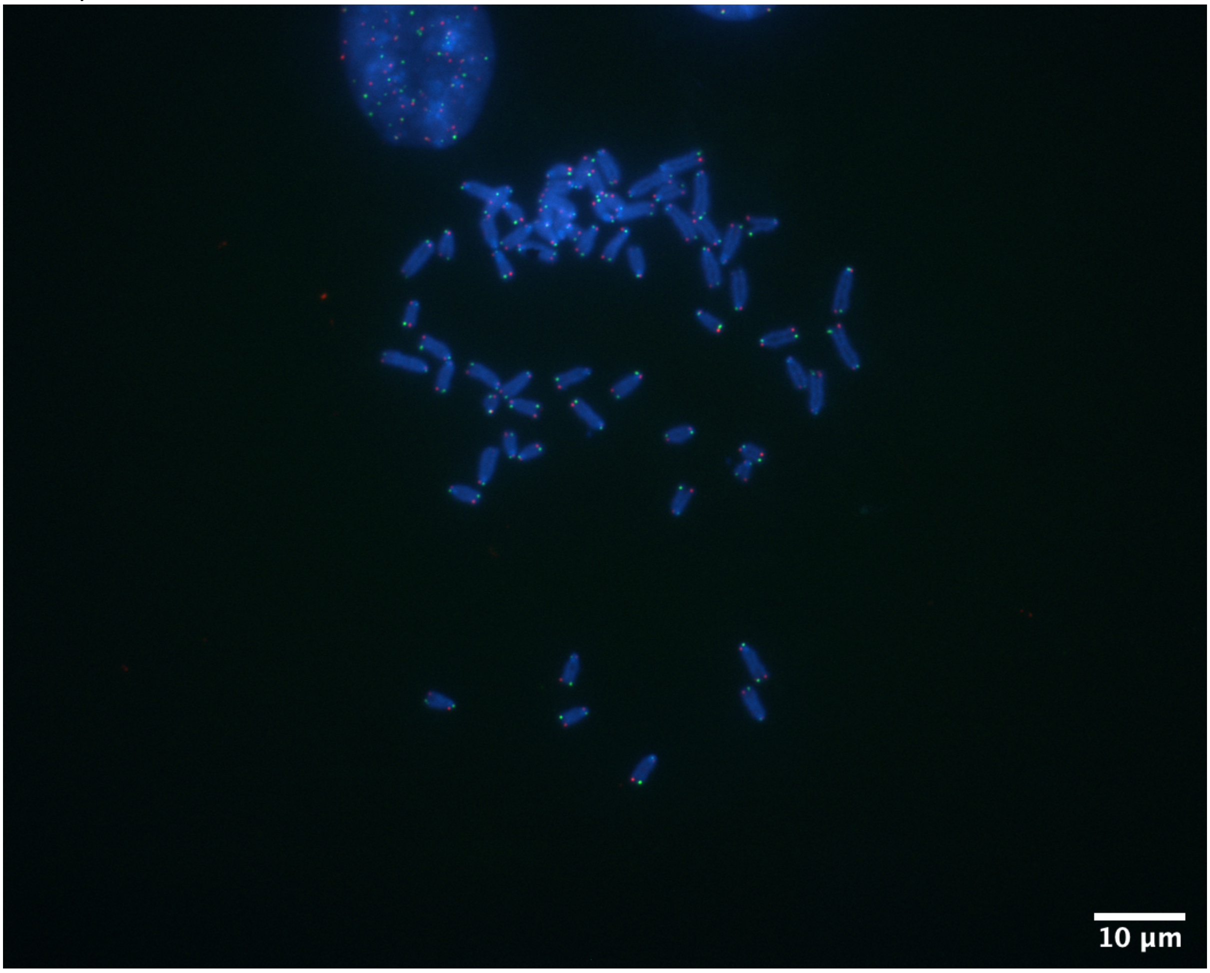

TRF2F/F Rosa-CreER^{T1} + TRF2-F120A + 4-OHT

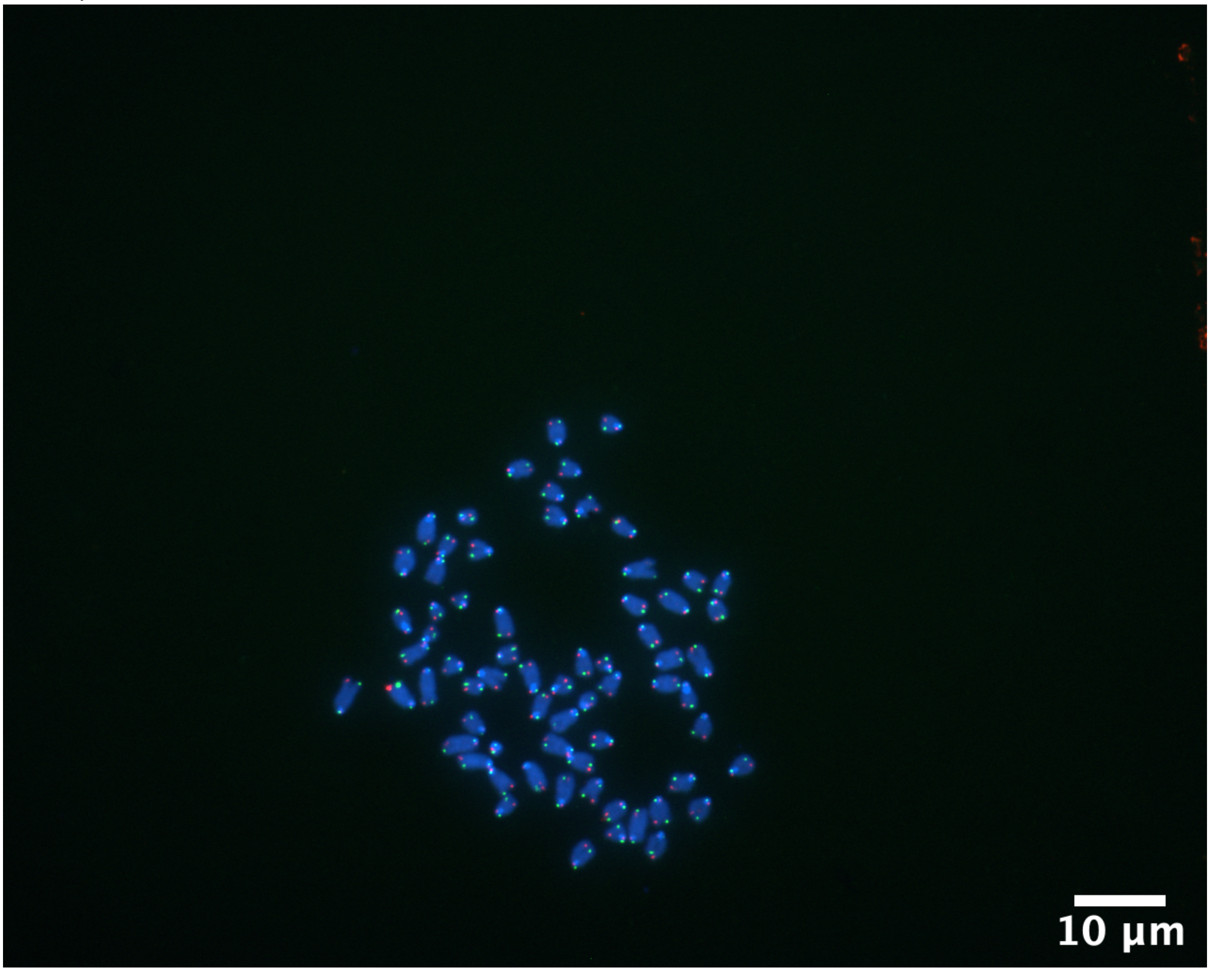

TRF2F/F Rosa-CreER^{T1} + TRF2-F120AΔiDDR + 4-OHT

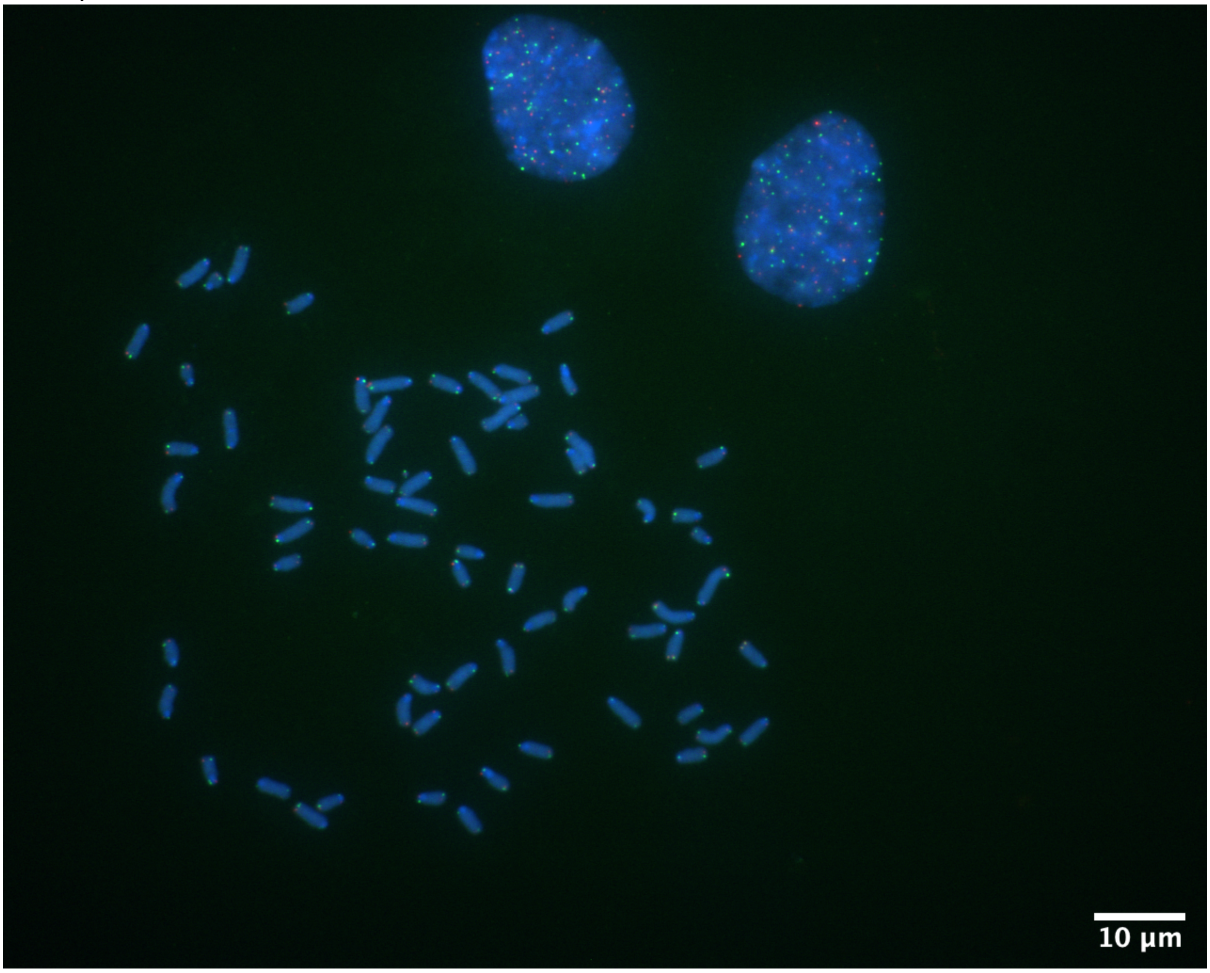

Supplement: Source Data Fig. 4 — Uncropped western blots. Uncropped scans of telomere overhang gels. Uncropped and unprocessed metaphases. [file 41594_2023_1072_MOESM9_ESM.pdf]
