# Supplementary material for: DNA-PK and the TRF2 iDDR inhibit MRN-initiated resection at leading-end telomeres
Source: Nat Struct Mol Biol. 2023 Aug 31;30(9):1346–56. doi: 10.1038/s41594-023-01072-x (PMC10497418; doi:10.1038/s41594-023-01072-x)

Source Data Fig.5

Fig.5a,b TRF2$^{F/F}$ Nbs1$^{F/+}$ +Cre

Native: 1 2 3 4 5 6 7 8

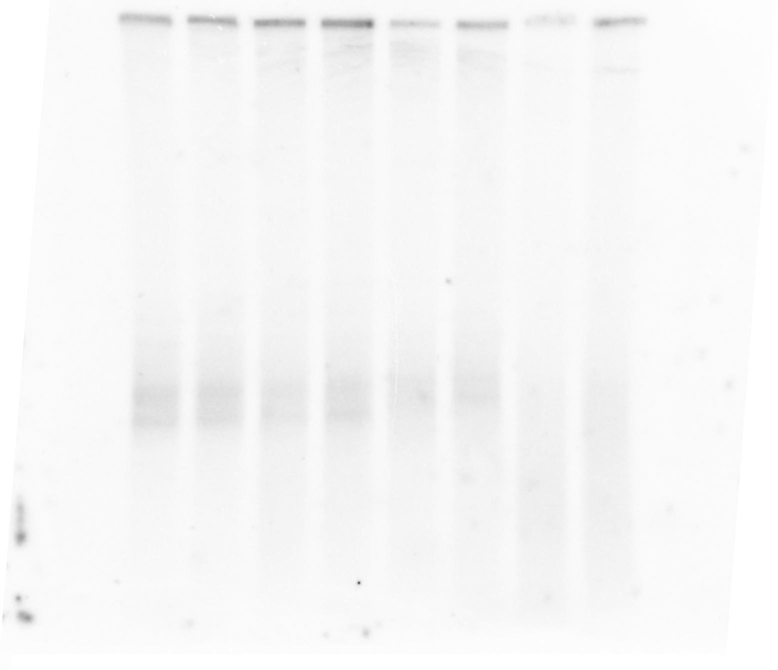

Denatured: 1 2 3 4 5 6 7 8

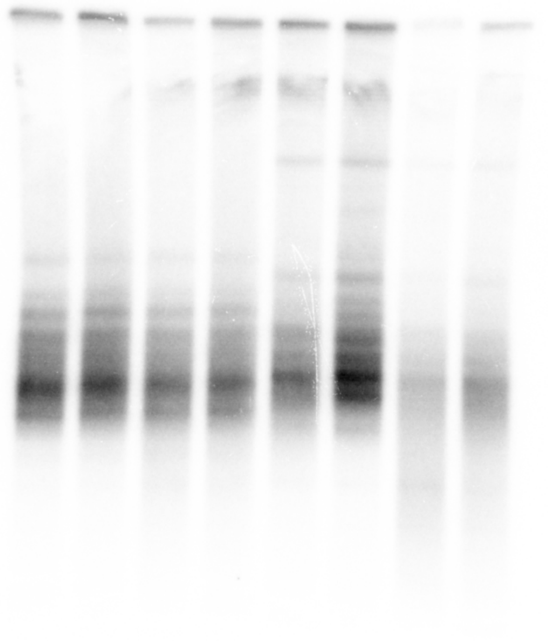

1. TRF2$^{F/F}$ Nbs1$^{F/+}$ +Cre + WT TRF2
2. TRF2$^{F/F}$ Nbs1$^{F/+}$ +Cre + ΔiDDR TRF2
3. TRF2$^{F/F}$ Nbs1$^{F/+}$ +Cre + F120A TRF2
4. TRF2$^{F/F}$ Nbs1$^{F/+}$ +Cre + F120A/ΔiDDR TRF2
5. /
6. /
7. /
8. /

Fig.5a,b TRF2$^{F/F}$ Nbs1$^{F/-}$ +Cre

Native: 1 2 3 4 5 6 7 8

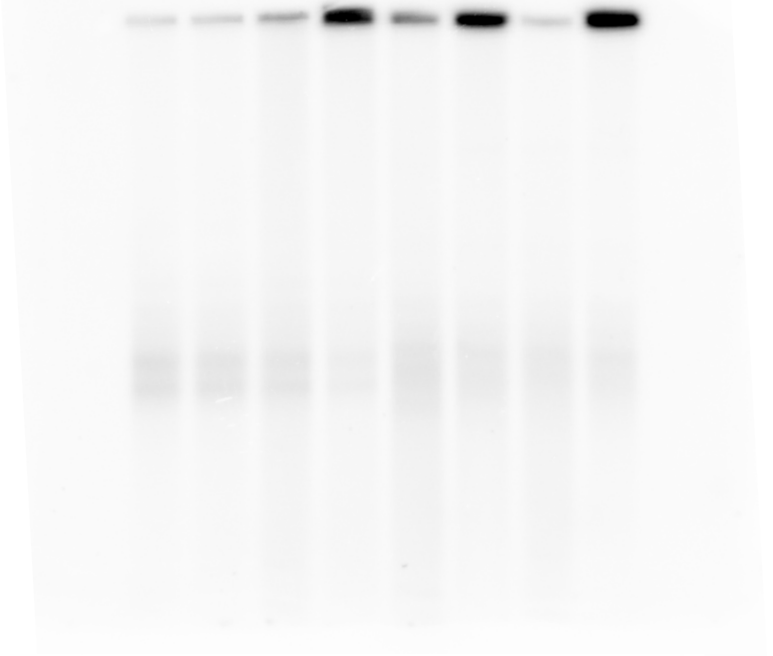

Denatured: 1 2 3 4 5 6 7 8

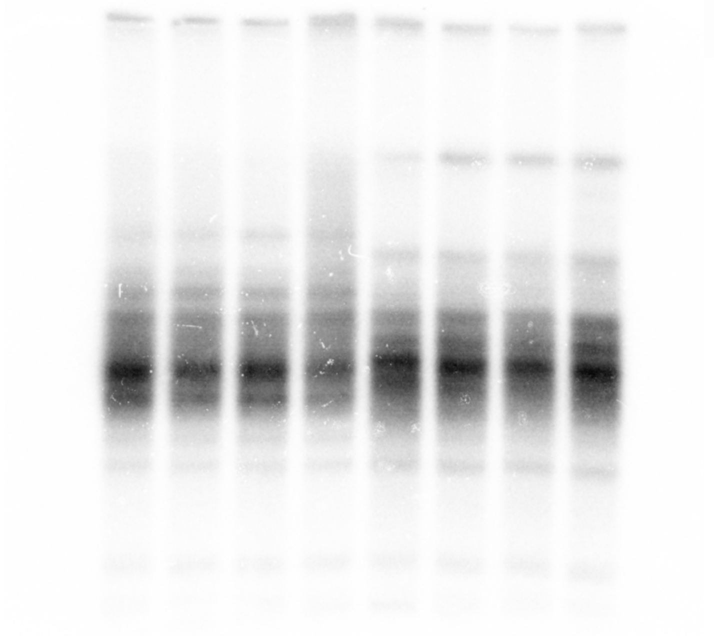

1. /
2. /
3. /
4. /
5. TRF2$^{F/F}$ Nbs1$^{F/-}$ +Cre + WT TRF2
6. TRF2$^{F/F}$ Nbs1$^{F/-}$ +Cre + ΔiDDR TRF2
7. TRF2$^{F/F}$ Nbs1$^{F/-}$ +Cre + F120A TRF2
8. TRF2$^{F/F}$ Nbs1$^{F/-}$ +Cre + F120A/ΔiDDR TRF2

Fig.5c, d
Blue: DAPI (DNA)
Green: Cy3-OO-(CCCTAA)$_3$ (Lagging-end telomeres)
Red: Alexa-647-(TTAGGG)$_3$ (Leading-end telomeres)

TRF2$^{F/F}$ Nbs1$^{F/+}$ +Cre +WT TRF2:

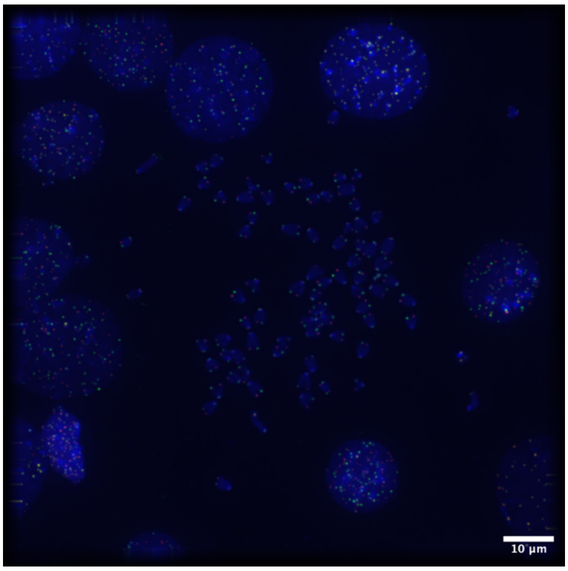

TRF2$^{F/F}$ Nbs1$^{F/+}$ +Cre +ΔiDDR TRF2:

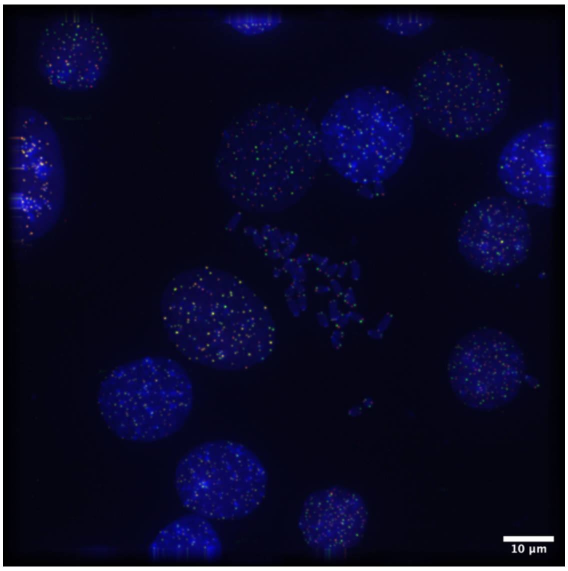

TRF2$^{F/F}$ Nbs1$^{F/+}$ +Cre +F120A TRF2:

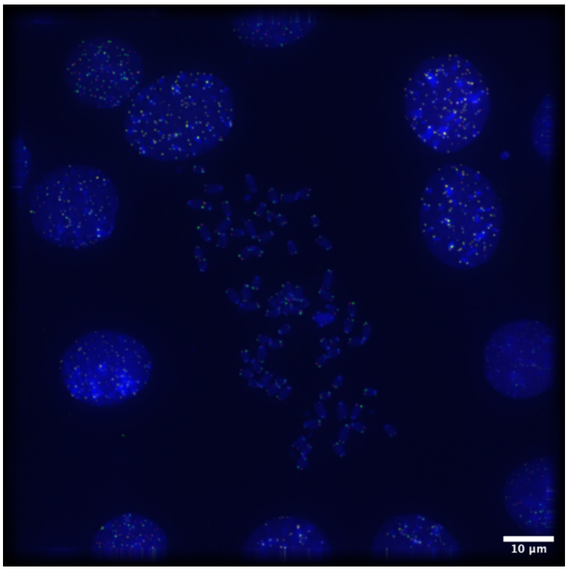

TRF2$^{F/F}$ Nbs1$^{F/+}$ +Cre +F120A/ΔiDDR TRF2:

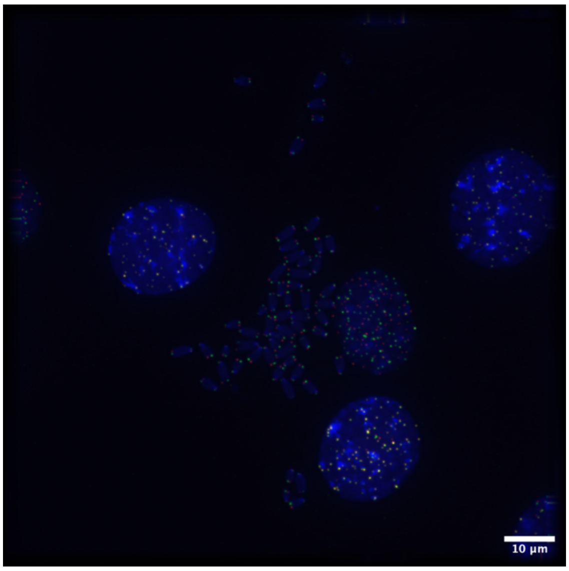

TRF2$^{F/F}$ Nbs1$^{F/-}$ +Cre +WT TRF2:

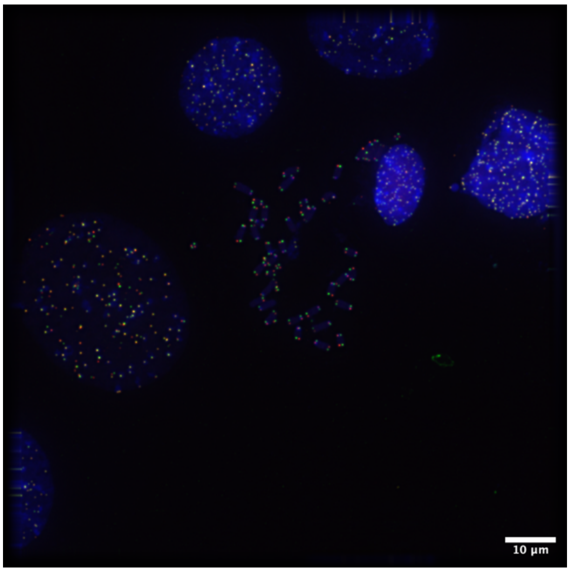

TRF2$^{F/F}$ Nbs1$^{F/-}$ +Cre +ΔiDDR TRF2:

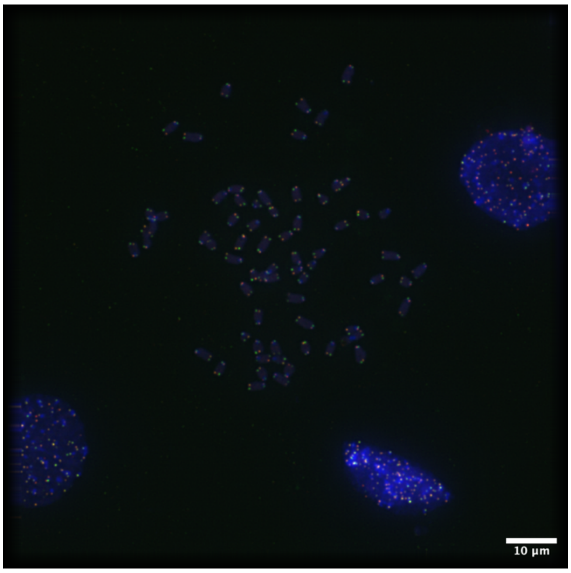

TRF2$^{F/F}$ Nbs1$^{F/-}$ +Cre +F120A TRF2:

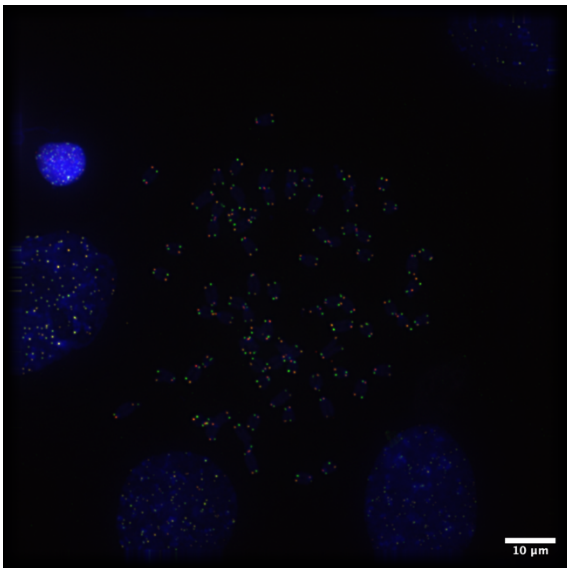

TRF2$^{F/F}$ Nbs1$^{F/-}$ +Cre +F120A/ΔiDDR TRF2:

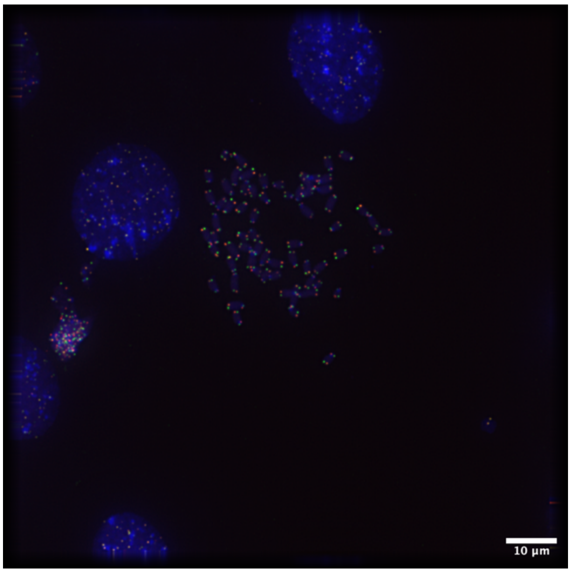

Supplement: Source Data Fig. 5 — Uncropped scans of telomere overhang gels. Uncropped and unprocessed metaphases. [file 41594_2023_1072_MOESM11_ESM.pdf]
