# Supplementary material for: DNA-PK and the TRF2 iDDR inhibit MRN-initiated resection at leading-end telomeres
Source: Nat Struct Mol Biol. 2023 Aug 31;30(9):1346–56. doi: 10.1038/s41594-023-01072-x (PMC10497418; doi:10.1038/s41594-023-01072-x)
Supplement: Source Data Fig. 6 — Uncropped scan of endonuclease and exonuclease assays. [file 41594_2023_1072_MOESM13_ESM.pdf]

Source Data Fig.6

Fig.6c

1 2 3 4 5 6 7 8 9 10 11 12 13 14 15 16

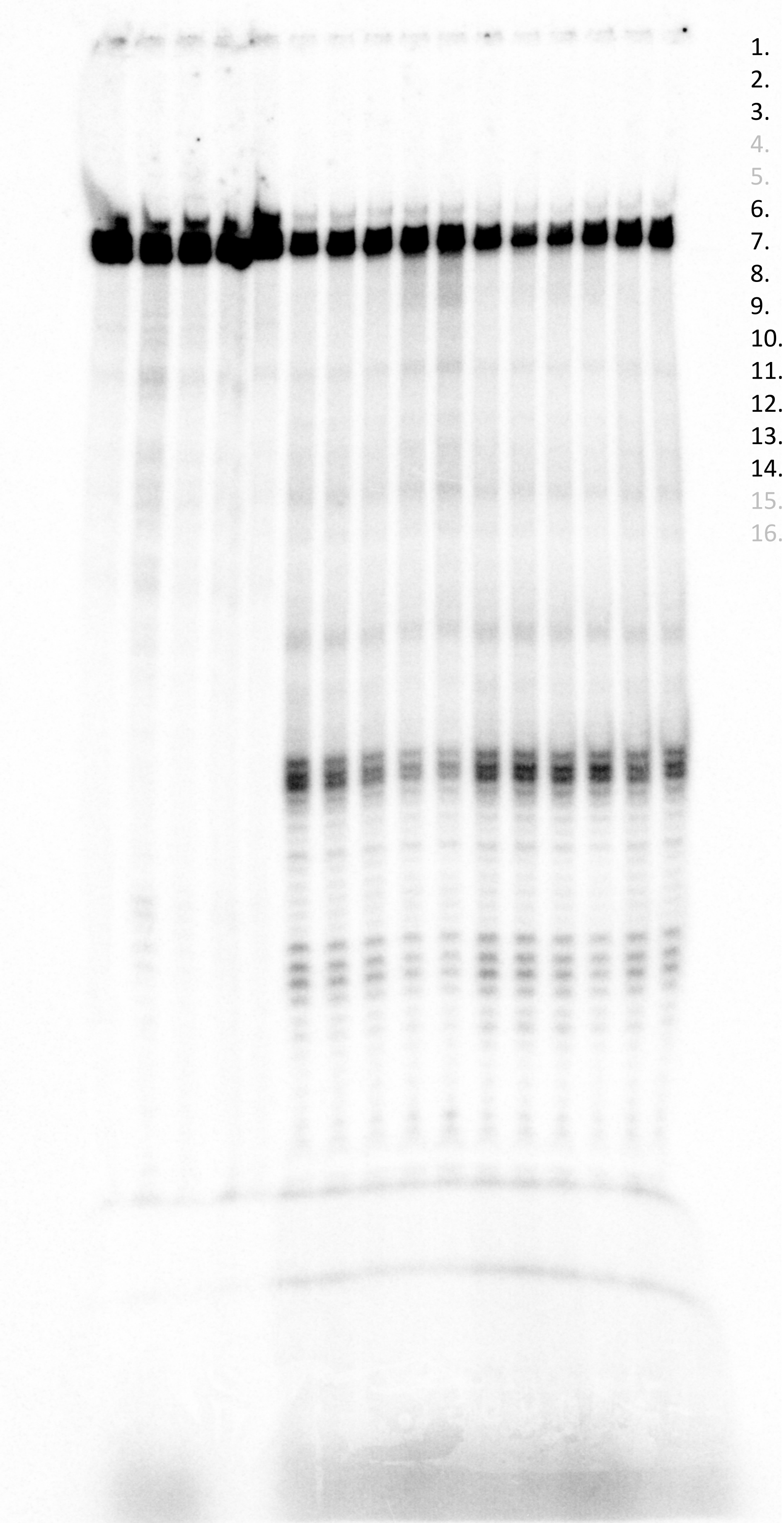

1. Buffer only
2. TRF2 wt only
3. TRF2 ΔiDDR only
4. /
5. /
6. MRN rxn
7. MRN rxn + 25nM TRF2
8. MRN rxn + 50nM TRF2
9. MRN rxn + 100nM TRF2
10. MRN rxn + 200nM TRF2
11. MRN rxn + 25nM TRF2ΔiDDR
12. MRN rxn + 50nM TRF2ΔiDDR
13. MRN rxn + 100nM TRF2ΔiDDR
14. MRN rxn + 200nM TRF2ΔiDDR
15. /
16. /

Fig.6e,f

1 2 3 4 5 6 7 8 9 10

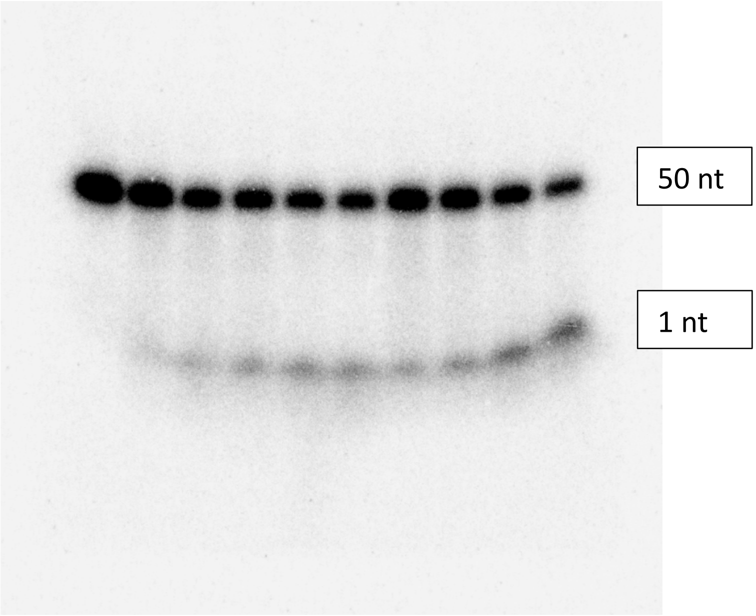

1. Buffer only
2. MRN rxn
3. MRN rxn + 50nM TRF2
4. MRN rxn + 100nM TRF2
5. MRN rxn + 200nM TRF2
6. MRN rxn + 400nM TRF2
7. MRN rxn + 50nM TRF2ΔiDDR
8. MRN rxn + 100nM TRF2ΔiDDR
9. MRN rxn + 200nM TRF2ΔiDDR
10. MRN rxn + 400nM TRF2ΔiDDR
