# Supplementary material for: DNA-PK and the TRF2 iDDR inhibit MRN-initiated resection at leading-end telomeres
Source: Nat Struct Mol Biol. 2023 Aug 31;30(9):1346–56. doi: 10.1038/s41594-023-01072-x (PMC10497418; doi:10.1038/s41594-023-01072-x)
Supplement: Source Data Extended Data Fig. 1 — Uncropped western blots. [file 41594_2023_1072_MOESM15_ESM.pdf]

Extended Data Fig1a

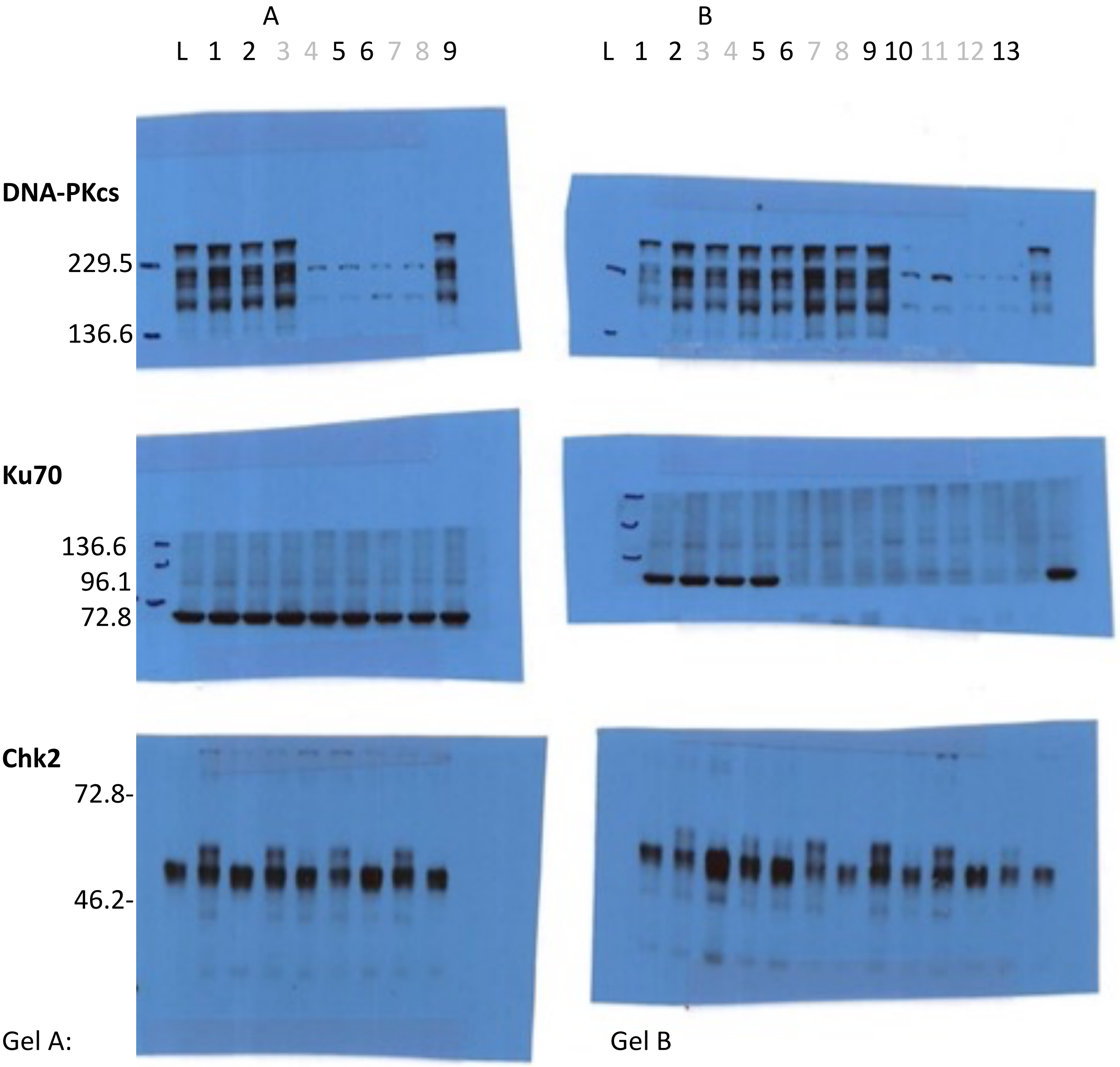

Gel A:

L: Ladder
1. Apollo$^{F/F}$ DNAPKcs$^{+/+}$ Ku70$^{+/+}$ no Cre
2. Apollo$^{F/F}$ DNAPKcs$^{+/+}$ Ku70$^{+/+}$ + Cre
3. /
4. /
5. Apollo$^{F/F}$ DNAPKcs$^{-/-}$ Ku70$^{+/+}$ no Cre
6. Apollo$^{F/F}$ DNAPKcs$^{-/-}$ Ku70$^{+/+}$ + Cre
7. /
8. /
9. Apollo$^{F/F}$ DNAPKcs$^{+/+}$ Ku70$^{+/+}$ no Cre

Gel B

L: Ladder
1. Apollo$^{F/F}$ DNAPKcs$^{+/+}$ Ku70$^{+/+}$ no Cre
2. Apollo$^{F/F}$ DNAPKcs$^{+/+}$ Ku70$^{+/+}$ + Cre
3. /
4. /
5. Apollo$^{F/F}$ DNAPKcs$^{+/+}$ Ku70$^{-/-}$ no Cre
6. Apollo$^{F/F}$ DNAPKcs$^{+/+}$ Ku70$^{-/-}$ + Cre
7. /
8. /
9. Apollo$^{F/F}$ DNAPKcs$^{-/-}$ Ku70$^{-/-}$ no Cre
10. Apollo$^{F/F}$ DNAPKcs$^{-/-}$ Ku70$^{-/-}$ + Cre
11. /
12. /
13. Apollo$^{F/F}$ DNAPKcs$^{+/+}$ Ku70$^{+/+}$ no Cre

Extended Data Fig1d

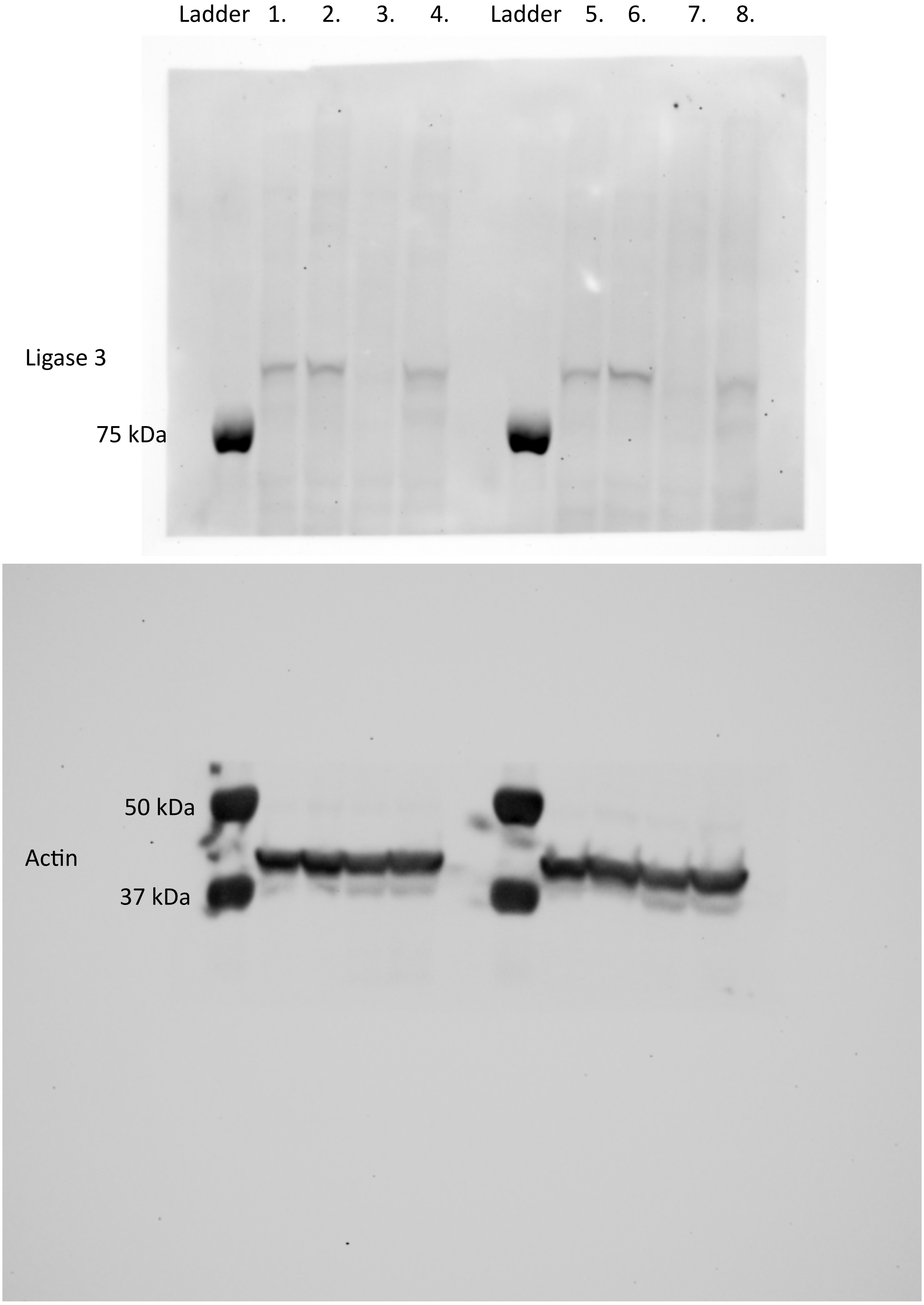

1. Apollo$^{F/F}$ + EV no Cre
2. Apollo$^{F/F}$ + EV + Cre
3. Apollo$^{F/F}$ + shRNA Lig3 + Cre
4. Apollo$^{F/F}$ + shRNA PolQ + Cre
5. Apollo$^{F/F}$ + EV no Cre
6. Apollo$^{F/F}$ + EV + Cre
7. Apollo$^{F/F}$ + shRNA Lig3 + Cre
8. Apollo$^{F/F}$ + shRNA PolQ + Cre
