# Supplementary material for: DNA-PK and the TRF2 iDDR inhibit MRN-initiated resection at leading-end telomeres
Source: Nat Struct Mol Biol. 2023 Aug 31;30(9):1346–56. doi: 10.1038/s41594-023-01072-x (PMC10497418; doi:10.1038/s41594-023-01072-x)
Supplement: Source Data Extended Data Fig. 2 — Uncropped scans of telomere overhang gels. Uncropped western blot. [file 41594_2023_1072_MOESM17_ESM.pdf]

ExtendedDataFig.2

ExtendedDataFig.2a,b: experiment I

Native: 1 2 3 4 5 6 7 8 9 10 11 12

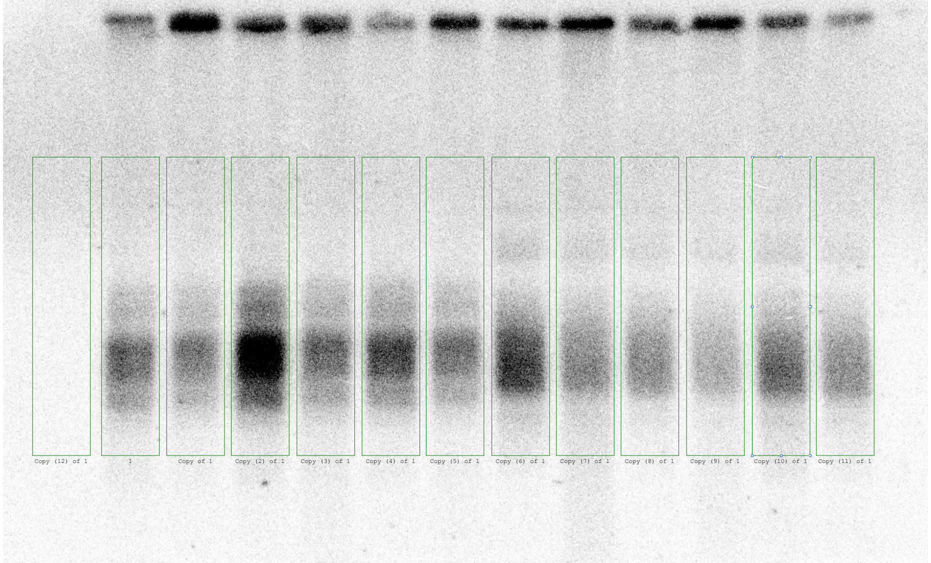

Denatured: 1 2 3 4 5 6 7 8 9 10 11 12

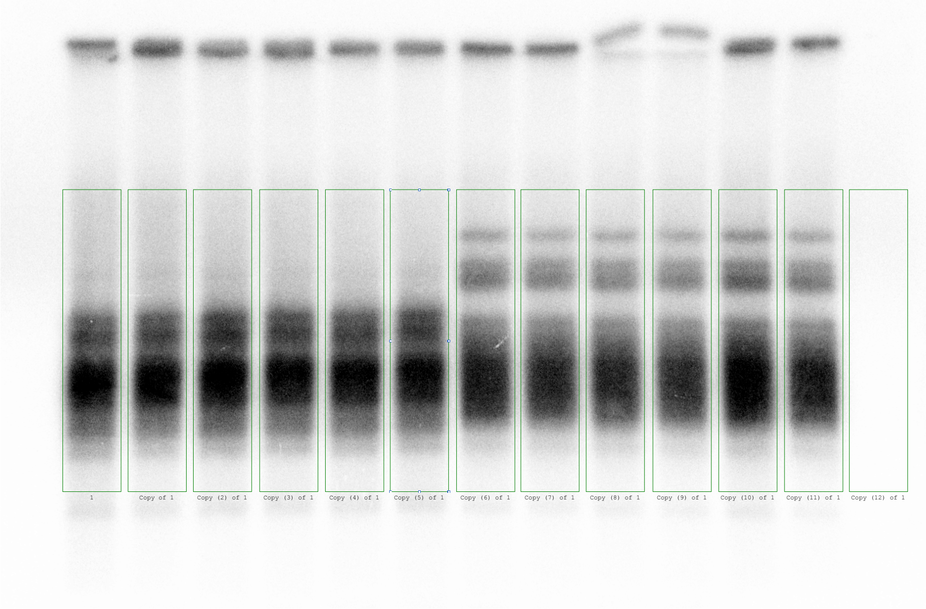

1. /
2. /
3. Apollo$^{F/F}$ Lig4$^{+/+}$ no Cre
4. Apollo$^{F/F}$ Lig4$^{+/+}$ + Cre
5. /
6. /
7. Apollo$^{F/F}$ Lig4$^{-/-}$ no Cre
8. Apollo$^{F/F}$ Lig4$^{-/-}$ + Cre
9. /
10. /
11. /
12. /

ExtendedDataFig.2d:

Ku70:

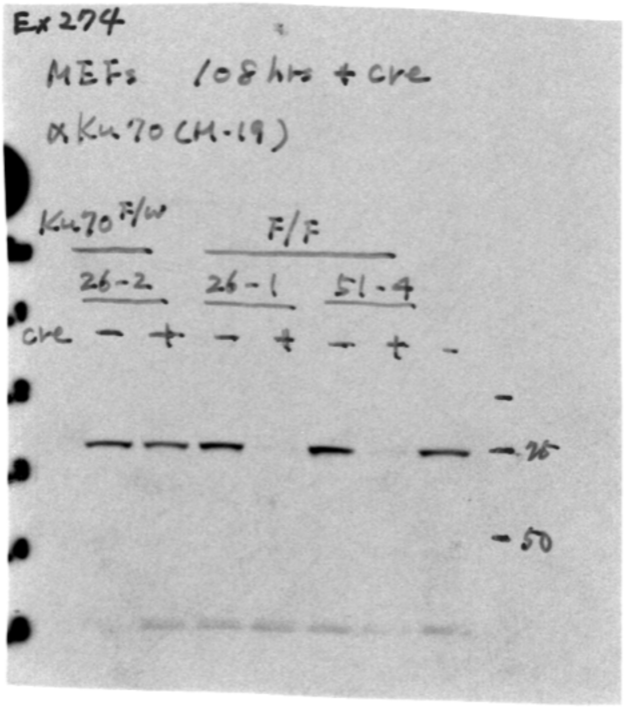

75 kDa

50 kDa

γ-tubulin

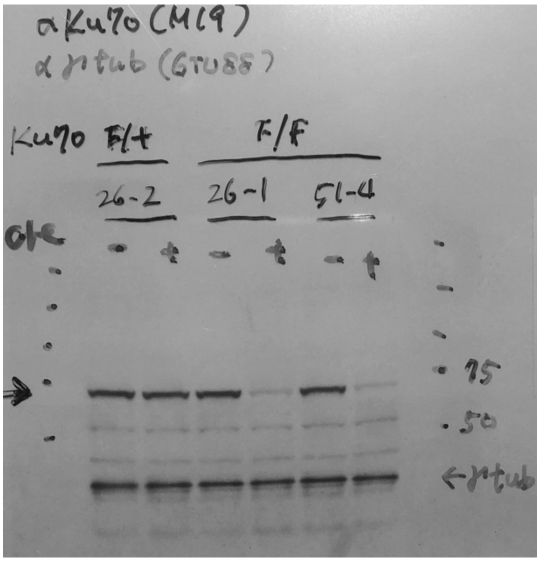

75 kDa
50 kDa

ExtendedDataFig.2f,g: experiment I

Native: 1 2 3 4 5 6 7 8 9 10 11 12 13

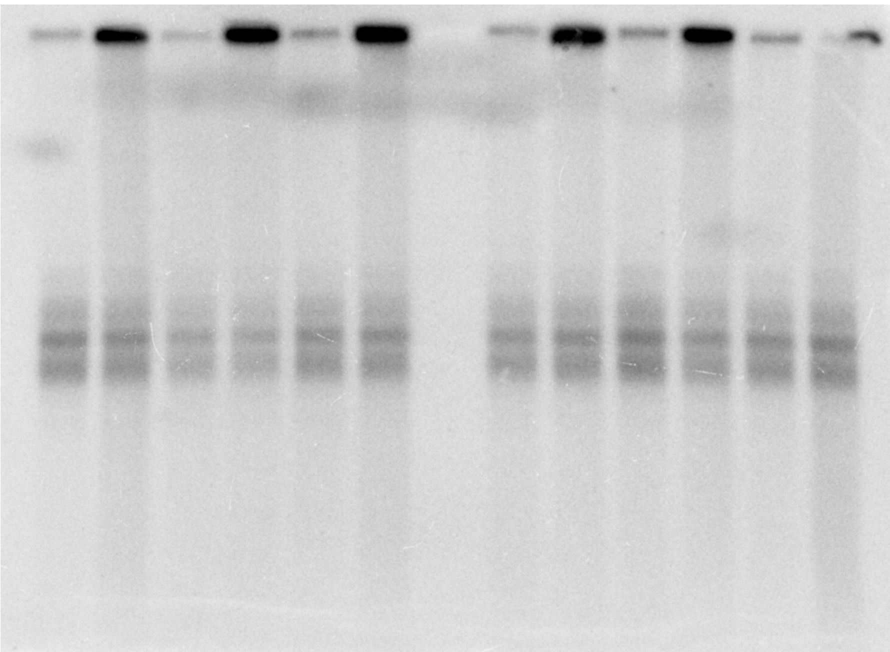

Denatured: 1 2 3 4 5 6 7 8 9 10 11 12 13

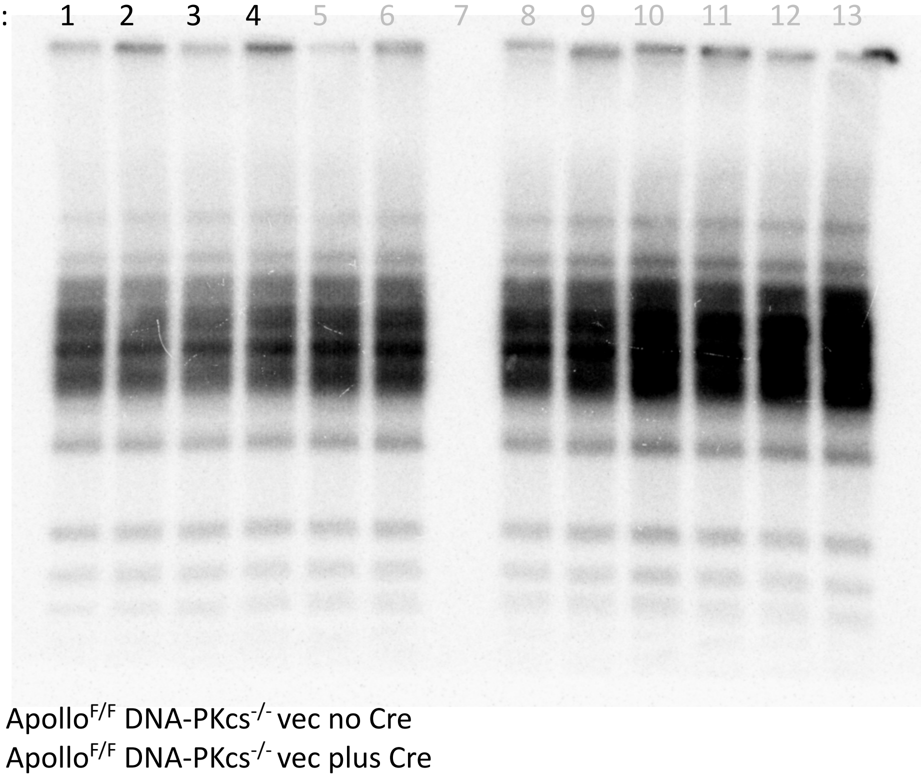

1. Apollo$^{F/F}$ DNA-PKcs$^{-/-}$ vec no Cre
2. Apollo$^{F/F}$ DNA-PKcs$^{-/-}$ vec plus Cre
3. Apollo$^{F/F}$ DNA-PKcs$^{-/-}$ shExo1 no Cre
4. Apollo$^{F/F}$ DNA-PKcs$^{-/-}$ shExo1 plus Cre
