# Supplementary material for: DNA-PK and the TRF2 iDDR inhibit MRN-initiated resection at leading-end telomeres
Source: Nat Struct Mol Biol. 2023 Aug 31;30(9):1346–56. doi: 10.1038/s41594-023-01072-x (PMC10497418; doi:10.1038/s41594-023-01072-x)

Extended Data Fig.3

Extended Data Fig.3b

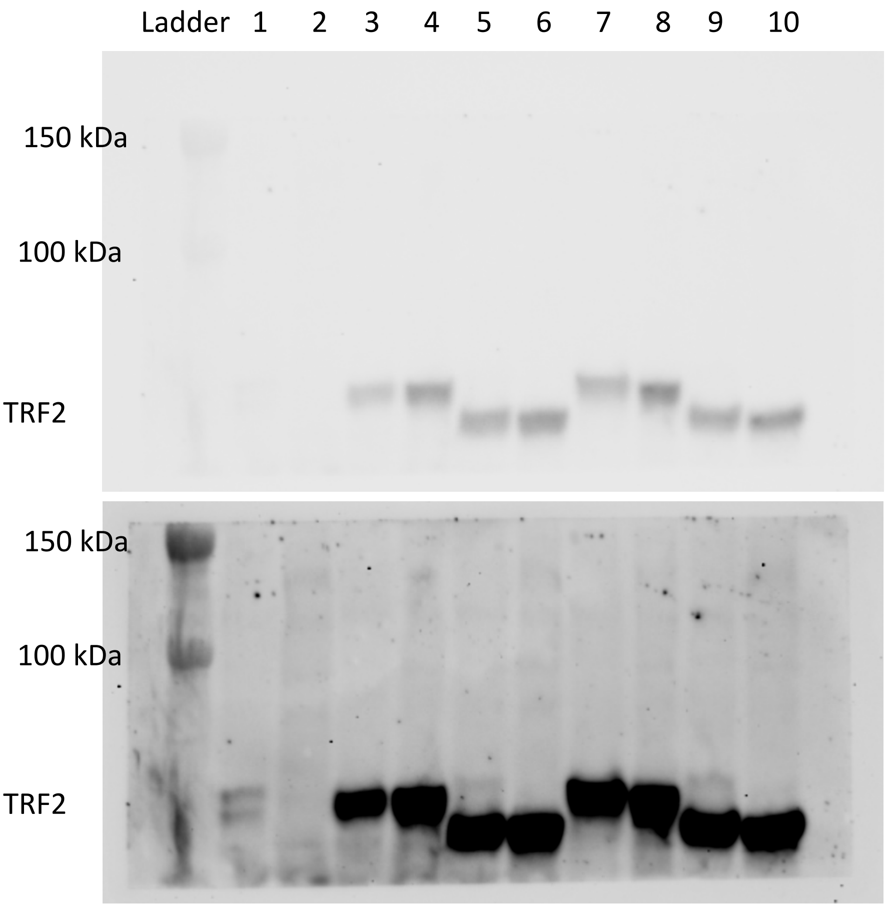

TRF2F/F Rosa-CreER^{T1} +

1. EV no Cre
2. EV + Cre
3. TRF2 no Cre
4. TRF2 + Cre
5. TRF2-ΔiDDR no Cre
6. TRF2-ΔiDDR + Cre
7. TRF2-F120A no Cre
8. TRF2-F120A + Cre
9. TRF2-F120AΔiDDR no Cre
10. TRF2-F120AΔiDDR + Cre

Extended Data Fig.3d:
Blue: DAPI (DNA)
Red: AlexaFluor 647 (γH2AX)
Green: Alexa Fluor 488-OO-(TTAGGG)$_3$ (telomeres)

TRF2F/F Rosa-CreER^{T1} + vec + 4-OHT:

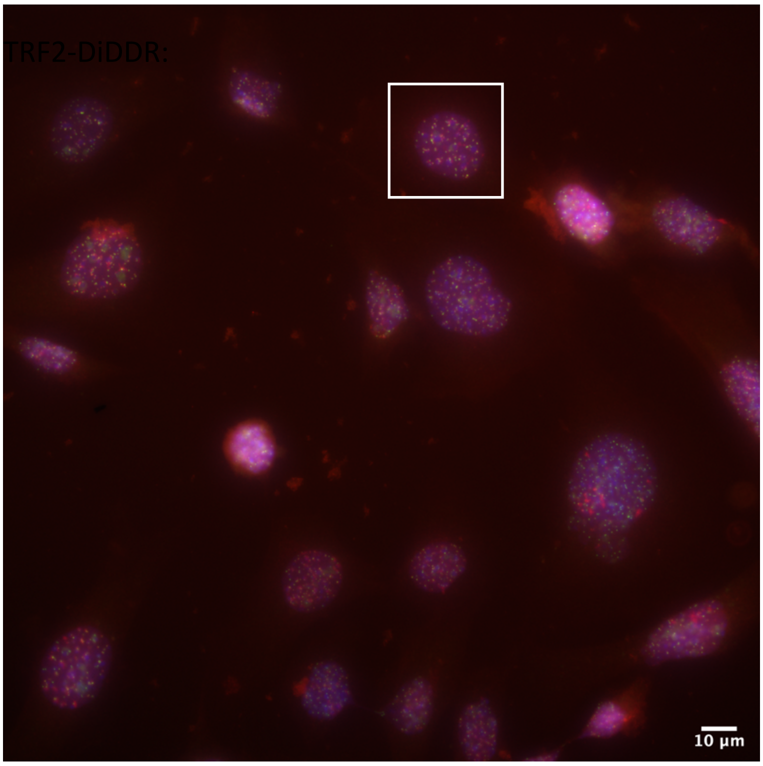

TRF2F/F Rosa-CreER^{T1} + TRF2-WT + 4-OHT:

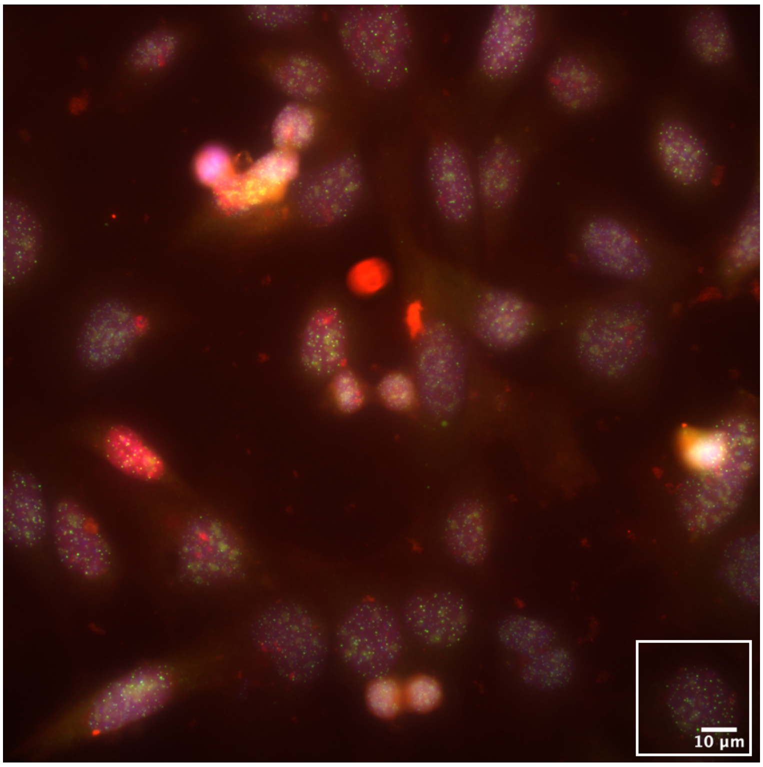

TRF2F/F Rosa-CreER^{T1} + TRF2-∆iDDR + 4-OHT (20221107_1):

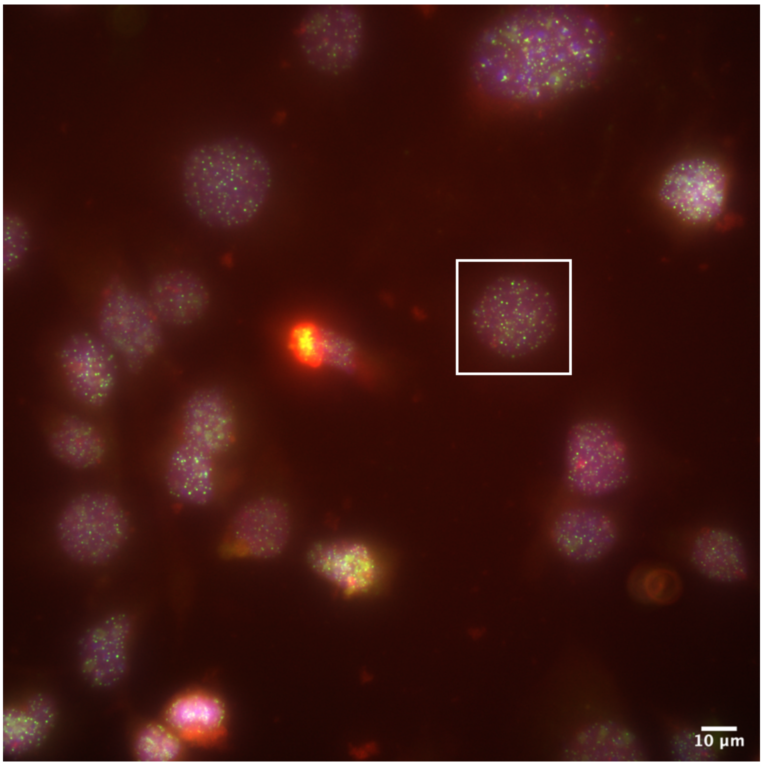

TRF2F/F Rosa-CreER^{T1} + TRF2-F120A + 4-OHT:

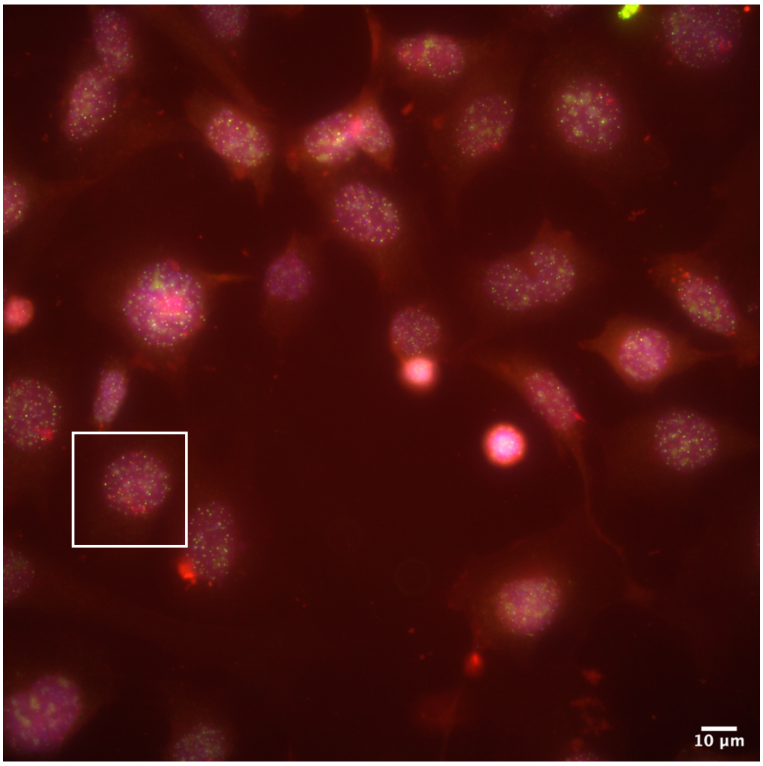

TRF2F/F Rosa-CreER^{T1} + TRF2-F120AΔiDDR + 4-OHT:

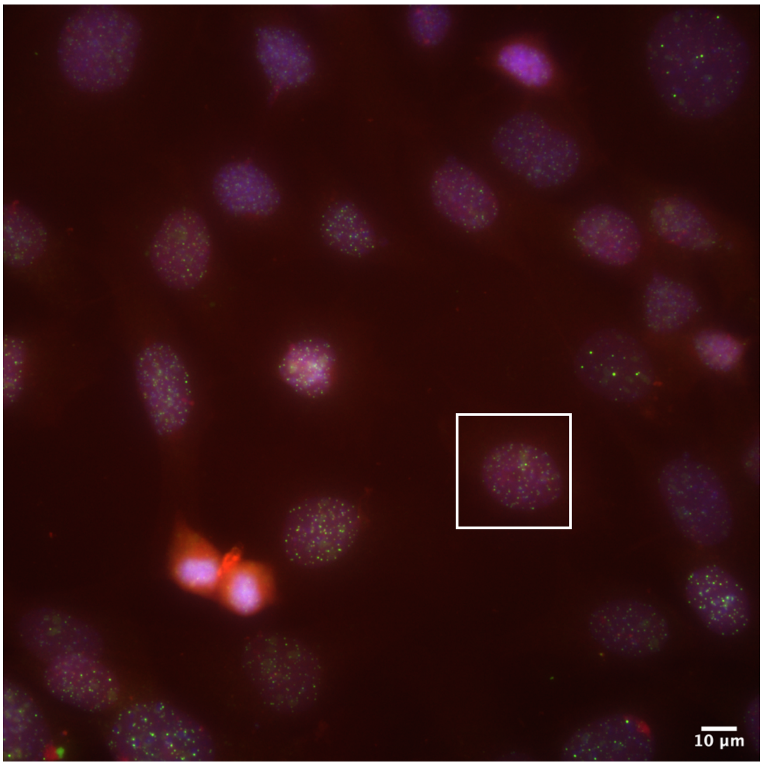

Supplement: Source Data Extended Data Fig. 3 — Uncropped western blots. Uncropped and unprocessed IF-FISH images. [file 41594_2023_1072_MOESM19_ESM.pdf]
