# Supplementary material for: DNA-PK and the TRF2 iDDR inhibit MRN-initiated resection at leading-end telomeres
Source: Nat Struct Mol Biol. 2023 Aug 31;30(9):1346–56. doi: 10.1038/s41594-023-01072-x (PMC10497418; doi:10.1038/s41594-023-01072-x)
Supplement: Source Data Extended Data Fig. 4 — Uncropped scans of telomere overhang gels. [file 41594_2023_1072_MOESM21_ESM.pdf]

Extended Data Fig. 4

Extended Data Fig. 4a:

Native: 1. 2. 3. 4. 5. 6. 7. 8. 9. 10. 11. 12. 13. 14.

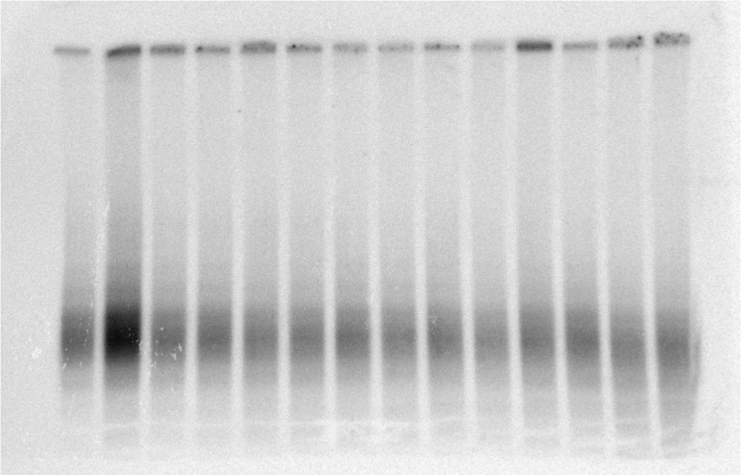

Denatured: 1. 2. 3. 4. 5. 6. 7. 8. 9. 10. 11. 12. 13. 14.

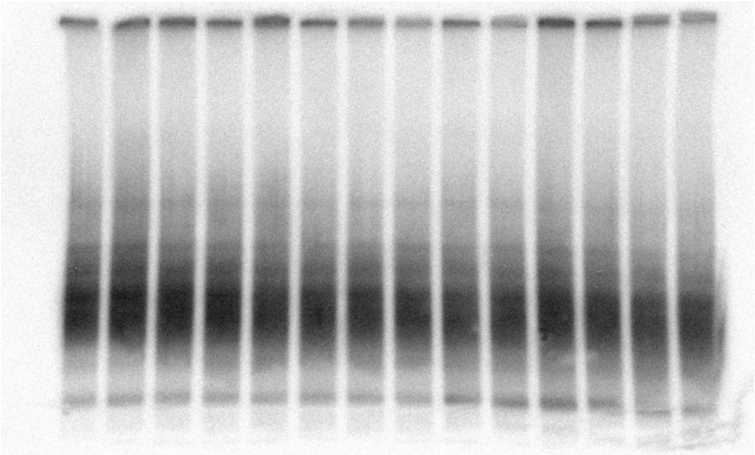

1. TRF2$^{F/F}$ 53BP1$^{-/-}$ Rosa-CreER^{T1} + EV no 4-OHT
2. TRF2$^{F/F}$ 53BP1$^{-/-}$ Rosa-CreER^{T1} + EV + 4-OHT
3. TRF2$^{F/F}$ 53BP1$^{-/-}$ Rosa-CreER^{T1} + TRF2-WT no 4-OHT
4. TRF2$^{F/F}$ 53BP1$^{-/-}$ Rosa-CreER^{T1} + TRF2-WT + 4-OHT
5. /
6. /
7. TRF2$^{F/F}$ 53BP1$^{-/-}$ Rosa-CreER^{T1} + TRF2-F120A no 4-OHT
8. TRF2$^{F/F}$ 53BP1$^{-/-}$ Rosa-CreER^{T1} + TRF2-F120A + 4-OHT
9. /
10. /
11. TRF2$^{F/F}$ 53BP1$^{-/-}$ Rosa-CreER^{T1} + TRF2-ΔiDDR no 4-OHT
12. TRF2$^{F/F}$ 53BP1$^{-/-}$ Rosa-CreER^{T1} + TRF2-ΔiDDR + 4-OHT
13. TRF2$^{F/F}$ 53BP1$^{-/-}$ Rosa-CreER^{T1} + TRF2-F120AΔiDDR no 4-OHT
14. TRF2$^{F/F}$ 53BP1$^{-/-}$ Rosa-CreER^{T1} + TRF2-F120AΔiDDR + 4-OHT

Extended Data Fig. 4b:

Native: 1 2 3 4

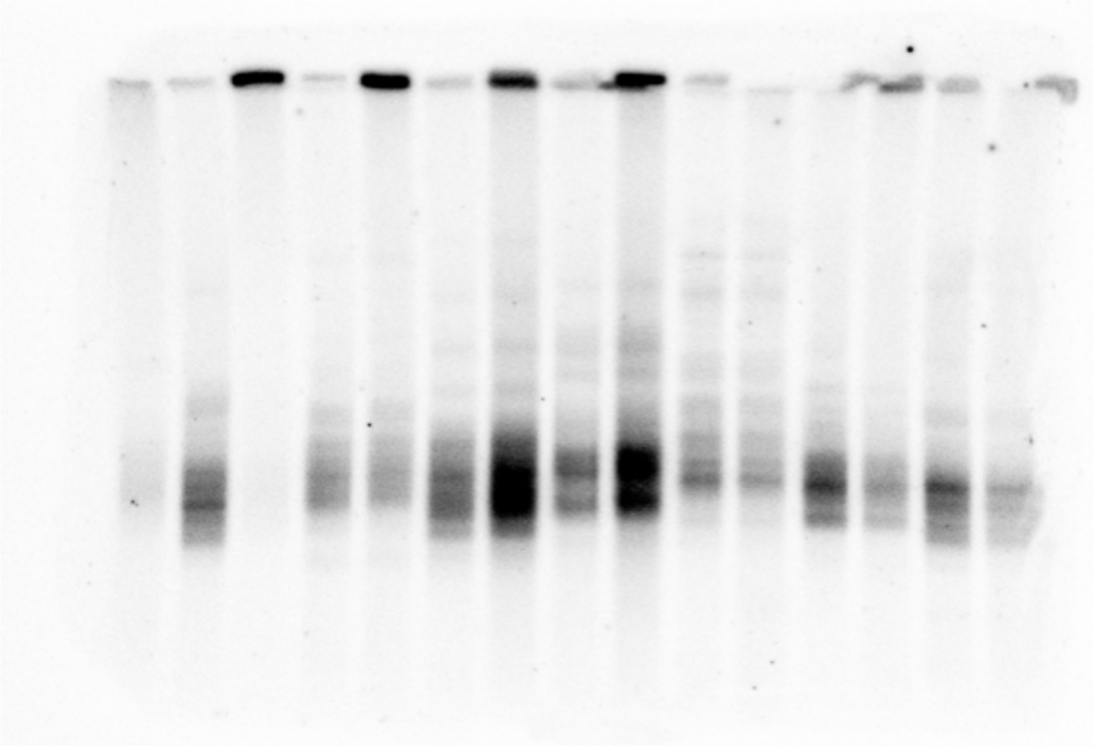

Denatured: 1 2 3 4

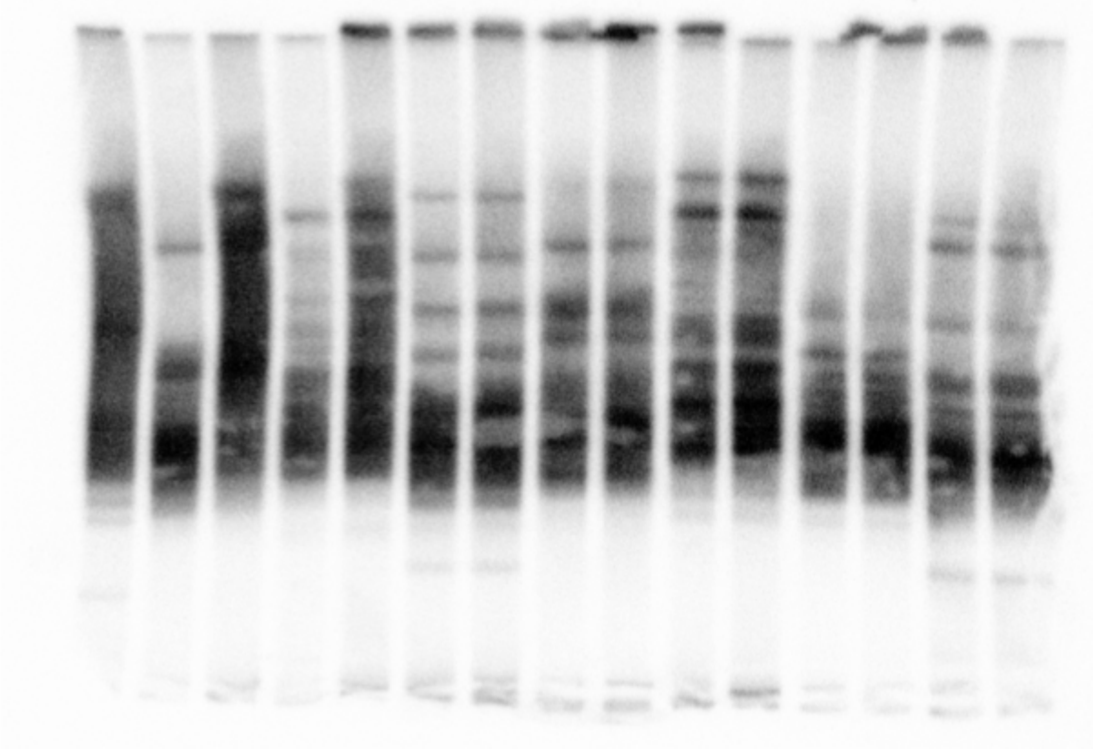

1. Apollo$^{F/F}$ 53BP1$^{+/+}$ no Cre
2. Apollo$^{F/F}$ 53BP1$^{+/+}$ + Cre
3. Apollo$^{F/F}$ 53BP1$^{-/-}$ no Cre
4. Apollo$^{F/F}$ 53BP1$^{-/-}$ + Cre
